# Supplementary material for: SARS-CoV-2 hijacks a cell damage response, which induces transcription of a more efficient Spike S-acyltransferase
Source: Nat Commun. 2023 Nov 11;14:7302. doi: 10.1038/s41467-023-43027-2 (PMC10640587; doi:10.1038/s41467-023-43027-2)

Supplementary Information for:

SARS-CoV-2 hijacks a cell damage response, which induces transcription of a more efficient Spike S-acyltransferase

Francisco S. Mesquita[1, #], Laurence Abrami[1,#], Lucie Bracq[1], Nattawadee Panyain[1] , Vincent Mercier[1,2], Béatrice Kunz[1], Audrey Chuat[1], Joana Carlevaro-Fita [1], Didier Trono[1], and F. Gisou van der Goot[1]

Supplementary Tables

Table S1

Common name	Identifier	Group	Name
Northern pike	A0A6Q2XEA9_ESOLU	fish Esociformes	Esox lucius
Brown trout	A0A674DFK2_SALTR	fish Salmoniformes	Salmo trutta
Seriola lalandi dorsalis/Yellowtail ray-finned fish	A0A3B4Y925_SERLL	fish Acanthomorphata	Seriola lalandi dorsalis
zebra mbuna	A0A3P9CQU4_9CICH	fish Acanthomorphata	Maylandia zebra
Rope fish	A0A8C4S801_ERPCA	fish Polypteriformes	Erpetoichthys calabaricus
Ghost shark	A0A4W3I3P3_CALMI	fish Chondrichthyes	Callorhinchus milii
Western clawed frog	A0A6I8QK71_XENTR	frog	Xenopus tropicalis
baker's yeast	YLR246W	yeast	Saccharomyces cerevisiae
Roundworm	C43H6.7b.1	worm	Caenorhabditis elegans
Leishan spiny toad	A0A8C5LWE9_9ANUR	frog	Leptobrachium leishanense
Duckbill platypus	F7EKK0_ORNAN	Monotremata	Ornithorhynchus anatinus
Goat	A0A452F7G6_CAPHI	artiodactyle	Capra hircus
Narwhal	A0A8C6FDZ5_MONMO	artiodactyle	Monodon monoceros
Sperm whale	A0A2Y9SYX1_PHYMC	artiodactyle	Physeter macrocephalus
Dog	A0A8I3PF12_CANLF	carnivore	Canis lupus familiaris
Red fox	A0A3Q7RZD7_VULVU	carnivore	Vulpes vulpes
Cat	A0A5F5XTX8_FELCA	carnivore	Felis catus
Lesser hedgehog tenrec	ENSETEP00000006408	echinops	Echinops telfairi
Greater horseshoe bat	A0A671DTP7_RHIFE	bat	Rhinolophus ferrumequinum
Little brown bat	G1PP97_MYOLU	bat	Myotis lucifugus

Golden hamster	ENSMAUP00000001611	rodent	Mesocricetus auratus
Rat	A0A8I6AR22_RAT	rodent	Rattus norvegicus
Steppe mouse	A0A8C6GNK5_MUSSI	rodent	Mus spicilegus
Human	ZDH20_HUMAN	human	Homo sapiens
Green monkey	A0A0D9RZN5_CHLSB	primate	Chlorocebus sabaeus
Panamanian white-faced capuchin	A0A2K5S968_CEBIM	primate	Cebus imitator
White-tufted-ear marmoset	F7I8C0_CALJA	primate	Callithrix jacchus
Western painted turtle	A0A8C3I713_CHRPI	turtle	Chrysemys picta bellii
Three-toed box turtle	A0A674JIK4_TERCA	turtle	Terrapene carolina triunguis
Goodes thornscrub tortoise	A0A8C4VHT4_9SAUR	turtle	Gopherus evgoodei
Kakapo/owl parrot owl parrot	A0A672UL97_STRHB	bird	Strigops habroptila
Chicken	A0A1D5P262_CHICK	bird	Gallus gallus
Tuatara	A0A8D0HET4_SPHPU	reptile	Sphenodon punctatus
American chameleon	G1KB70_ANOCA	reptile	Anolis carolinensis
Eastern brown snake	A0A670Y5U8_PSETE	reptile	Pseudonaja textilis
Asian bonytongue / arowana	A0A8C9SXY1_SCLFO	fish Osteoglossocephala	Scleropages formosus
Transparent sea squirt	Q1RPW1_CIOIN	Ciona	Ciona intestinalis
Fruit fly	FBpp0086152	fly	Drosophila melanogaster
Dianchi golden-line fish	A0A672PWU6_SINGR	fish Ostariophysi	Sinocyclocheilus grahami
Common carp	A0A8C1DMS8_CYPCA	fish Ostariophysi	Cyprinus carpio
Goldfish	ENSCARP00000107484	fish Ostariophysi	Carassius auratus
denticle herring	A0A8C3ZFP0_9TELE	fish Clupei	Denticeps clupeoides
Atlantic herring	ENSCHAP00000003299	fish Clupei	Clupea harengus
Northern pike	A0A3P8XWZ8_ESOLU	fish Esociformes	Esox lucius
Coho salmon	A0A8C7N188_ONCKI	fish Salmoniformes	Oncorhynchus kisutch
Rainbow trout	A0A8C7QR01_ONCMY	fish Salmoniformes	Oncorhynchus mykiss
bicolor damselfish	A0A3B5B3M0_9TELE	fish Acanthomorphata	Stegastes partitus
European seabass	ENSDLAP00005082700	fish Acanthomorphata	Dicentrarchus labrax

Table S1 – Full name and correspondent identifiers of the Species' common names depicted in Fig. 1k.

Table S2.

5 ' end positions determined by 5'RACE	Number of clones retrieved	position in relation to start site (-bp)	primer coverage	%	Sum % in fig S1D
22,039,980	2	6671	all	3.8	
22,038,728	1	5419	all	1.9	5.7
22,036,135	1	2826	all	1.9	
22,036,132	1	2823	all	1.9	3.8
22,035,430	2	2121	3,4,5	3.8	
22,035,237	2	1928	3,4,5	3.8	7.5
22,033,993	1	684	4,5	1.9	
22,033,509	1	200	5	1.9	
22,033,508	4	199	5	7.5	
22,033,486	2	177	5	3.8	
22,033,485	2	176	5	3.8	1.9
22,033,473	11	164	5	20.8	
22,033,438	1	129	coding	1.9	
22,033,422	2	113	coding	3.8	
22,033,422	7	113	coding	13.2	
22,033,416	7	107	coding	13.2	
22,033,407	3	98	coding	5.7	
22,033,382	2	73	coding	3.8	37.7
22,033,377	1	68	coding	1.9	43.4

Table S2 – Summary of 5'RACE analysis – with: positions of 5 ' ends determined by 5'RACE sequencing the number of clones retrieved for each end, the position of each 5'end in relation to the annotated human start site (Human version hg19- http://genome.ucsc.edu), the coverage or whether the sequence can be detected by the primers shown in Supplementary data Fig. 1D, and the percentages of clones determined for different coverage-groups depicted in **Supplementary data Fig. 1d.**

Supplementary Figures and Figure Legends

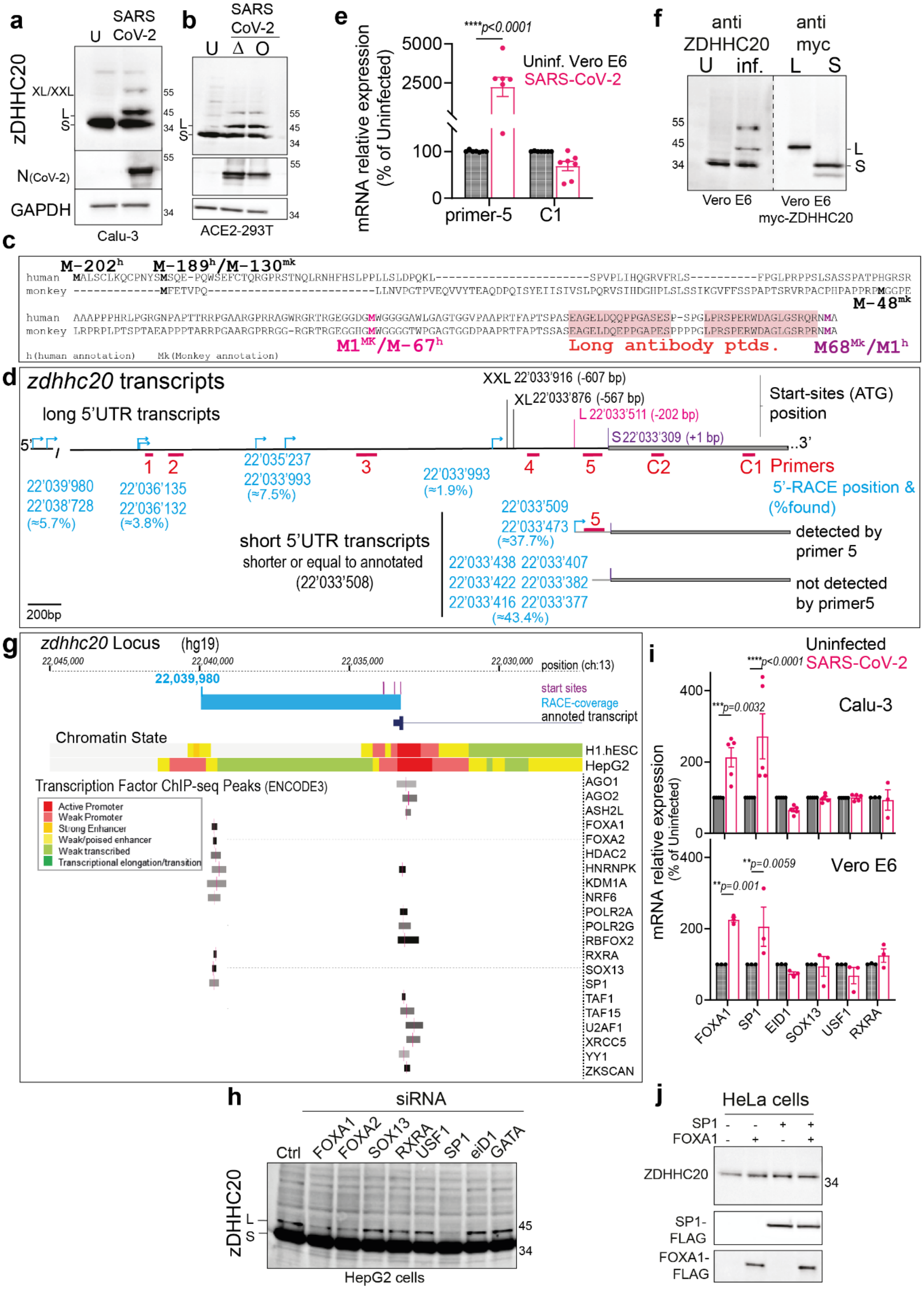

Supplementary Figure 1

a. WB of ZDHHC20, N, GAPDH on Calu-3 uninfected (U) or infected 24h with SARS-CoV-2, MOI=0.1. **b.** WB as in **a** of HEK293T-TMPRSS2-ACE2 uninfected (U) or infected 24 h with SARS-CoV-2 strain B.1 (left), or SARS-CoV-2 Delta B1.617.2 or Omicron BA.1 (right). **c.** Alignment of N-terminal amino acids of ZDHHC20 in human (UCSC genome browser - version hg19), and Green Monkey Chlorocebus Sabaeus (A0A0D9RZN5). Methionine (H-human or Mk-monkey) are annotated. $M1^{H}/M68^{Mk}$ in purple, $M1^{MK}/M\text{-}67^{H}$ in magenta others in black. Peptides used to generate rabbit antibodies against Long ZDHHC20 are in orange. **d.** Representation of the 5'UTR from *zdhhc20* transcripts. Long, transcripts (larger than); Short (smaller/equivalent) to annotated 5'UTR. 5' ends obtained by 5'RACE and abundance in blue (see also Table S2). QPCR primers in *zdhhc20* locus used in Fig 1 (red). Transcription start sites are indicated. Small transcripts are further classified regarding their detection by primer 5 **e.** mRNA quantification using primers probing for different locations in z*dhhc20* transcripts (coding region: C1 or in 5'UTR: primer-5) in Vero E6 cells treated as in **a**. **f.** WB of Vero E6 extracts: (left) uninfected (U) or infected as in **a** and probed with anti-ZDHHC20 (all) antibody; or (right) transfected with myc-ZDHHC20 (short-S or long-L) for 24 h and probed with anti-myc antibody. **g.** Overview of UCSC genome browser at *zdhhc20* locus and zoom in transcription start site (TSS) (Human version hg19). Two custom tracks and three UCSC genome browser tracks are shown: 1) start-sites (ATGs) indicates *zdhhc20* in-frame ATG codons; 2) 5'-RACE Sequence describes the genomic region amplified by 5'-RACE technique; 3) Transcript annotation corresponds to GENCODE Genes track (version V40lift37); 4) Chromatin state segmentation data from ENCODE consortia for two different cell lines (H1.hESC and HepG2) 5) Transcription Factor (TF) CHIP-seq Peaks track shows transcription factor (TF) binding sites for HepG2 cell line based on ChIP-seq experiments from ENCODE. Only TF with a minimum score range of 300 are shown. The level of peak enrichment is represented with the darkness of the item, and the vertical pink bar marks the point-source of the peak. **h.** WB of ZDHHC20 on HEPG2 cell extracts transfected 72 hours with siRNA targeting the indicated TFs. **i.** mRNA quantification of TFs in infected Calu-3 or Vero E6 cells. **j.** WB of ZDHHC20 and Flag on Hela extracts transfected 24 hours with plasmids expressing SP1-Flag or FOXA1-Flag. For all $*P < 0.05$, $**P < 0.01$, $***P < 0.001$ and $****P < 0.0001$, source data are provided as a Source Data file within Supplementary information (entire blot scans).

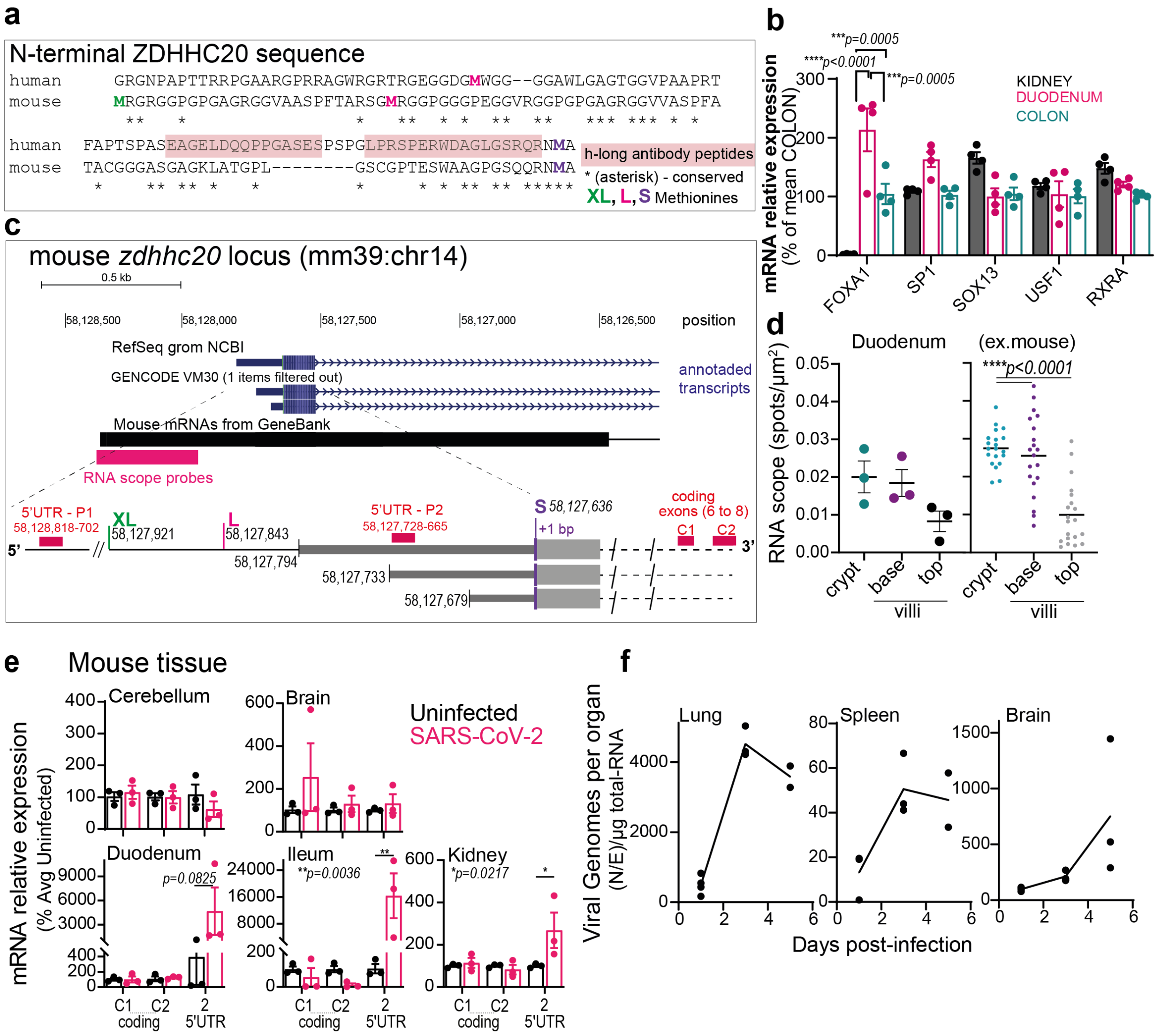

Supplementary Figure 2

a. Alignment of N-terminal amino acids of ZDHHC20 in human (genome browser: hg19), and Mouse (GRCm39/mm39). Peptides used to generate rabbit antibodies against human ZDHHC20^{Long} are highlighted in orange. In frame methionines (coloured) and conserved residues (*-asterisks) are indicated **b.** mRNA quantification in mouse kidney, duodenum and colon tissues, using primers for the indicated TFs. Results are mean + SEM and each dot represents one of 4 independent mice. *P* values were obtained by two-way ANOVA with Tukey's multiple comparison. **c.** Overview of murine *zdhhc20* locus (Mouse GRCm39/mm39) from UCSC genome browser https://genome.ucsc.edu) showing tracks corresponding to

transcripts from NCBI RNA reference sequences-Refseq and GENCODE (version VM30) (blue) and coverage of publicly available mouse *zdhhc20* mRNA sequences from Genbank (black). The position of *zdhhc20* RNA scope probes used in **Fig 2b** (pink) and QPCR primers (red) used in **Fig 2d** are indicated. Insets show the position of in-frame ATG codons at transcription start sites coding for XL (green), Long (magenta) and Short ZDHHC20 forms with the correspondent N-terminal extension depicted below. **d.** Average (left) and representative (one mouse) quantification of *zDHHC20* RNAscope spots on different parts (crypt, base, top) of the mouse duodenum**.** Average results (left) are mean + SEM and each dot represents one of 3 independent mice, whereas representative data (right) depicts mean values and corresponding distribution of RNA scope spots per randomly selected field-of-views (n = 20) for one representative mouse. *P* values were obtained by One-way ANOVA with Tukey's multiple comparison. **e.** mRNA quantification in different murine tissues (human ACE2 transgenic mice) uninfected or infected intranasally with 10^3-10^4 (plaque forming units – PFU) of SARS-CoV-2 (5-6 days post infection). Extracted mRNA was analysed using primers for different locations in z*dhhc20* transcripts (coding region (C1, C2) or the 5' UTR-2 depicted in **c. f.** Quantification of viral genome copies (N and E RNA) in different infected murine tissues harvested at the indicated times as in **e.** For all $*P < 0.05$, $**P < 0.01$, $***P < 0.001$ and $****P < 0.0001$. Source data are provided as a Source Data file and within Supplementary information (entire blot scans).

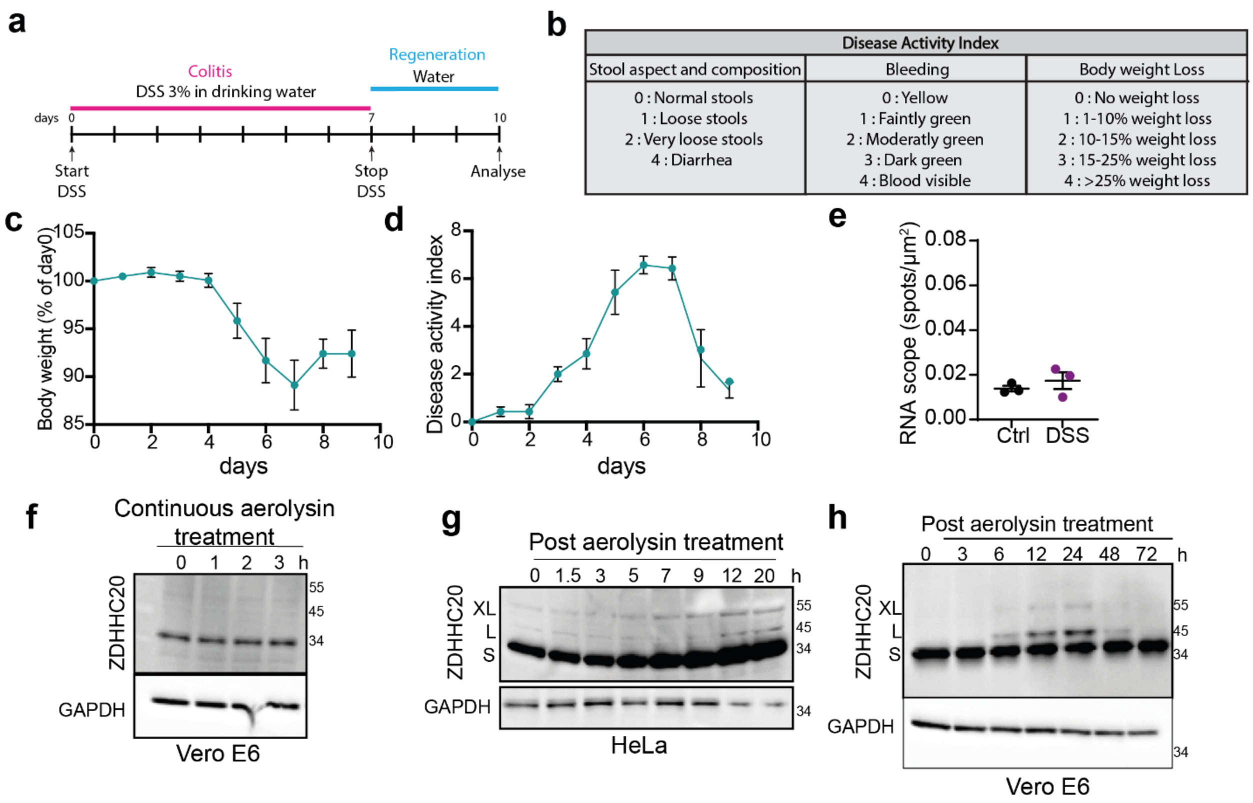

Supplementary Figure 3

a-d. DSS-induced colitis model. 8-weeks C56BL6/J male were given 3% Dextran-Sulfate-Sodium in the drinking water for 7 days, then switched to regular drinking water for 3 days and allowed to recover. **a**. Experimental scheme of DSS-induced colitis. **b-d**. Disease activity Index (DAI) scoring was performed daily including body weight loss (d), stool aspect and Occult blood in feces. Values are mean ± SEM of n=7 mice. **e.** Quantification of ZDHHC20 RNA scope spots on mouse colon from control or DSS-treated mice (7 days treatment plus 3 days recovery). Results are mean + SEM of n = 3 independent mice per condition **f.** WB analysis of ZDHHC20 expression in Vero E6 cells treated continuously for the indicated times with 10 ng/ml of proaerolysin at 37°C. **g.h.** WB of ZDHHC20 expression from, **g**-HeLa or, **h**-Vero E6 cell extracts treated 1 h with 10 ng/ml of proaerolysin at 37°C, washed and further incubated at 37°C for indicated time-points. ZDHHC20 S, L and XL forms are indicated and GAPDH was used as loading control. Source data are provided as a Source Data file within Supplementary information (entire blot scans).

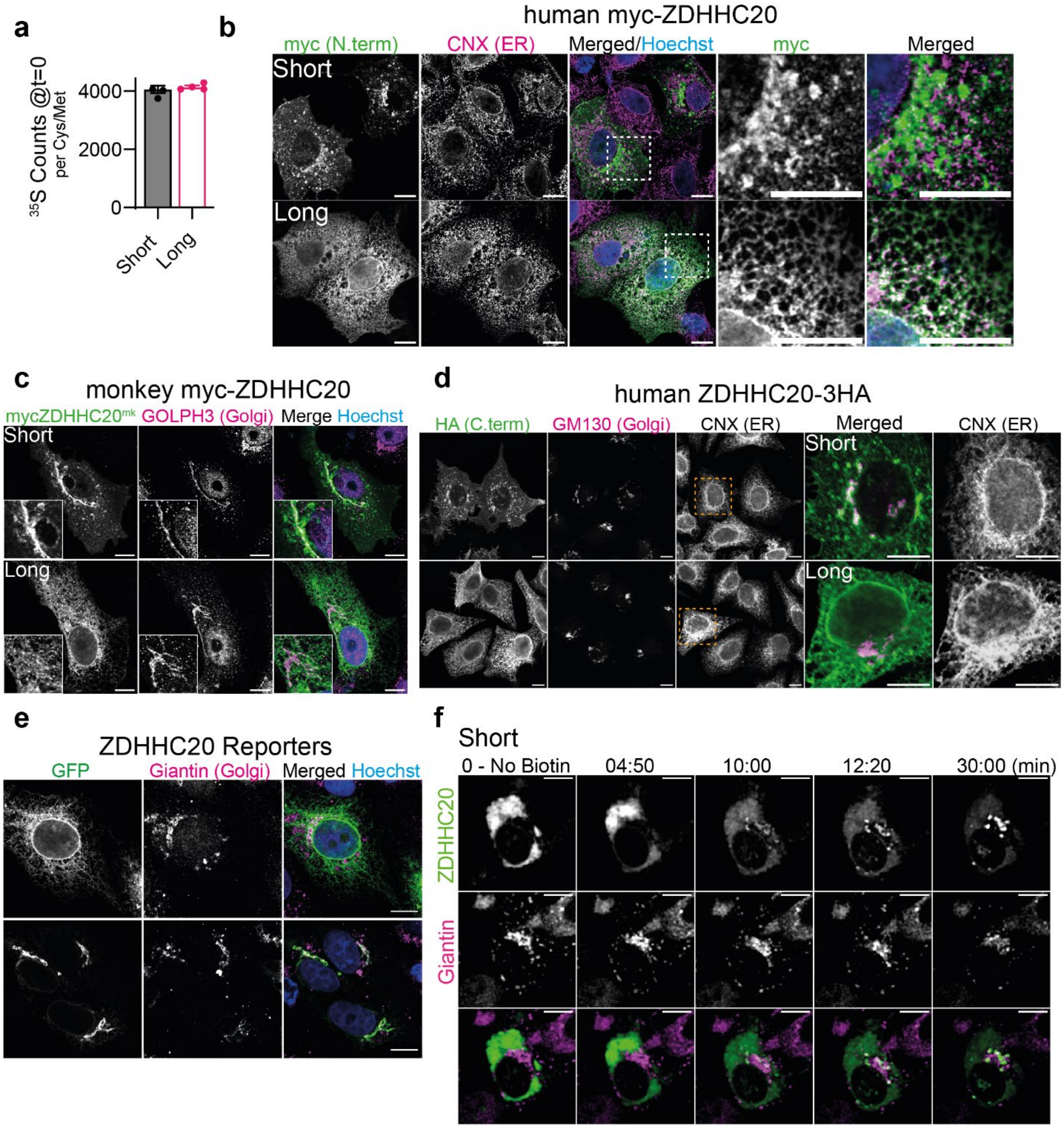

Supplementary Figure 4

a. Hela cells expressing myc-ZDHHC20 (Short or Long) metabolically labelled with 35S-Met/Cys for 20 min. 35S incorporation in myc-ZDHHC20 immunoprecipitation fractions were normalized by the number of Cys/Met residues per ZDHHC20 (20 for short and 21 for Long) and are mean ± SD of n = 4 independent experiments. **b.** IF of Vero E6 expressing myc-ZDHHC20 $^{\textbf{Short}}$ or myc-ZDHHC20$^{\textbf{Long}}$ labelled for myc, the ER marker Calnexin (CNX), and

nuclear-stained with Hoechst. Scale bar: 10µm. **c.** IF of Vero E6 expressing myc-ZDHHC20 $^{\mathbf{Short}}$ or myc-ZDHHC20^{Long} (Green Monkey Chlorocebus Sabaeus-A0A0D9RZN5 sequence) labelled for myc and the Golgi marker (GOLPH3), scale bar: 10 µm. **d.** IF of HeLa cells expressing C-terminally tagged ZDHHC20$^{\mathbf{Short}}$ -3HA or ZDHHC20^{Long} -3HA labelled for HA, Golgi marker GM130 and ER marker CNX, scale bar: 10µm. **e.** IF of Vero E6 expressing ZDHHC20^{Short}- or ZDHHC20^{Long}-reporters labelled for Golgi marker Giantin and nuclear-stained with Hoechst, scale-bar: 10 µm. **f.** Time-lapse confocal microscopy images of Vero E6 co-expressing (24 h) the Golgi marker Scarlet-Giantin, ZDHHC20^{short}-RUSH-GFP reporter and an ER-resident hook. Synchronized trafficking was monitored upon D-biotin addition (after T_0 see methods, **Fig. 4k** and **movies S1-2**). Source data are provided as a Source Data file.

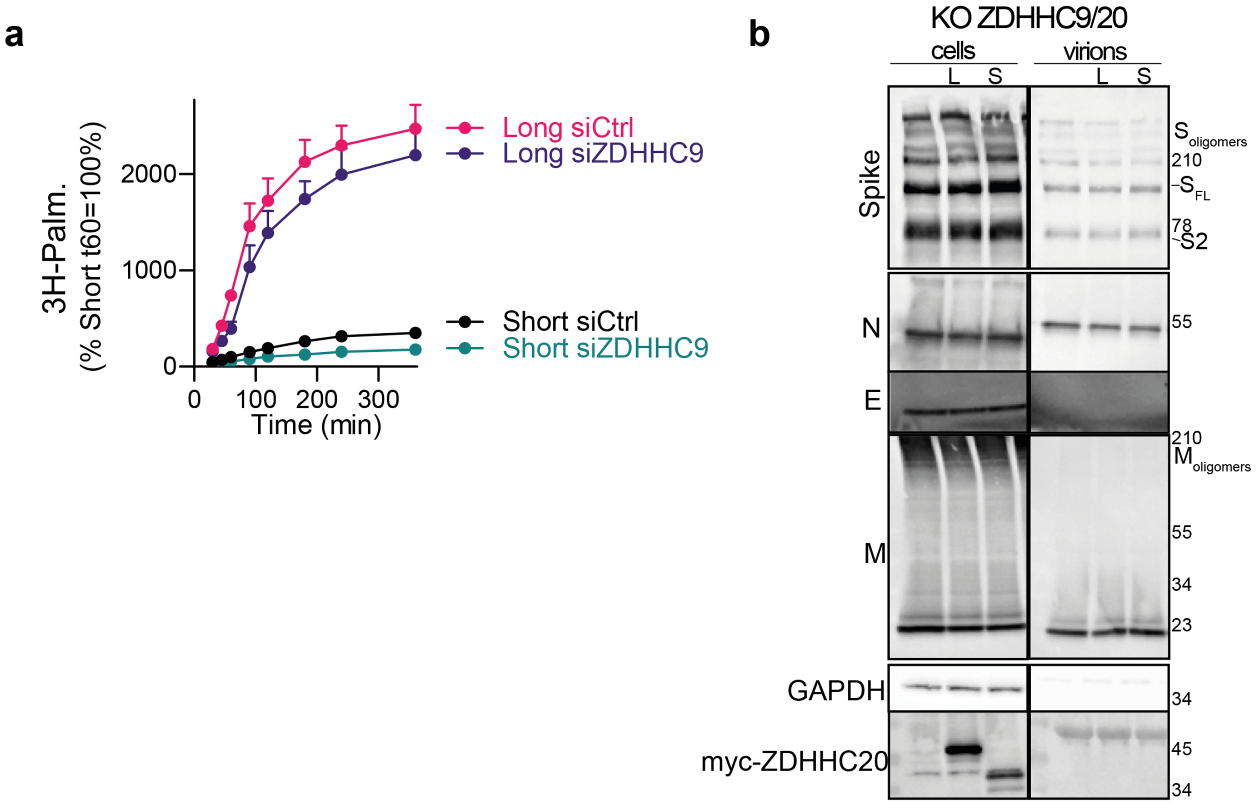

Supplementary Figure 5

a. Spike-HA incorporated 3H-palmitic acid normalized to T=60min in Vero E6 KO for ZDHHC20 with or without 3 days siRNA for ZDHHC9 recomplemented with ZDHHC20$^{\textbf{Short}}$ or ZDHHC20$^{\textbf{Long}}$ with half amount of DNA (eq.protein). **b.** WB of Spike, N, E, M, GAPDH, myc-ZDHHC20 on Vero E6 KO ZDHHC9/20 cell extracts (cells) transfected 24 h with control (Ctrl), ZDHHC20^{Short} (S) or ZDHHC20^{Long} (L) expressing plasmids, infected 24h with SARS-CoV-2, MOI:0.1, or on virions extracts collected from the corresponding cells. Source data are provided as a Source Data file within Supplementary information (entire blot scans).

Supplementary methods

- Code used for RNAscope quantification in Fig 2, 3 and Supplementary fig 3

* = CODE DESCRIPTION =
 * This script will apply a Laplacian of Gaussian filtered followed by a local maxima detector in order to locate
 * RNAScope spots in fluorescent images. The script works with an arbitrary number of channels, provided as a list.
 *
 * == MATERIALS & METHODS ==
 * RNAScope spots are identified using a Laplacian of Gaussian[1] filter (LoG) followed by a 2D local maximum finder.
 * In summary, annotated regions are imported from QuPath into ImageJ[2] and run through a median filter (radius=1.5 px)
 * in order to remove Poisson noise.
 * The diffraction-limited spots are then enhanced using a LoG filter (sigma=1.0 px). The resulting image is run through
 * ImageJ's local maximum finder algorithm with a channel-dependent tolerance value[3].
 * The local maxima points are then reimported into QuPath for each channel.
 *
 * REFERENCES
 * ----------
 * [1] https://imagescience.org/meijering/software/featurej/laplacian/
 * [2] Schneider, C. A., Rasband, W. S., & Eliceiri, K. W. (2012). NIH Image to ImageJ: 25 years of image analysis. Nature Methods, 9(7), 671–675. doi:10.1038/nmeth.2089
 * [3] https://github.com/imagej/ImageJ/blob/master/ij/plugin/filter/MaximumFinder.java
 *
 * == INPUTS ==
 * A single or multichannel fluorescence image with annotations. This script will run on all annotations
 * The user should specify the channel names to use, as well as the prominence values for each channel.
 * Higher prominence values will find fewer spots, as it represents how much of a difference the peaks should have with their
 * local background.
 *
 * == OUTPUTS ==
 * After running the code, mRNA spots will be shown Points, in the color of the channel.
 * Two new measurements "RNAScope CHANNEL Spots" and RNASctop CHANNEL Density are also appended to each annotation
 *
 * = DEPENDENCIES =
 * This script makes use of the ImageScience library at https://imagescience.org/meijering/software/imagescience/

```
 *
 * = INSTALLATION =
 * You must download 'imagescience.jar' and place it into your QuPath extensions directory
 * https://imagescience.org/meijering/software/imagescience/
 *
 * = AUTHOR INFORMATION =
 * Code written by Olivier Burri, EPFL - SV - PTECH - BIOP
 * for Lucie Bracq, Van Der Goot Lab
 * Last update: 20230314
 *
 * = COPYRIGHT =
 * © All rights reserved. ECOLE POLYTECHNIQUE FEDERALE DE LAUSANNE,
Switzerland, BioImaging and Optics Platform (BIOP), 2023
```

Supplementary Data Source – Entire Blot Scans

Figure 1

1a

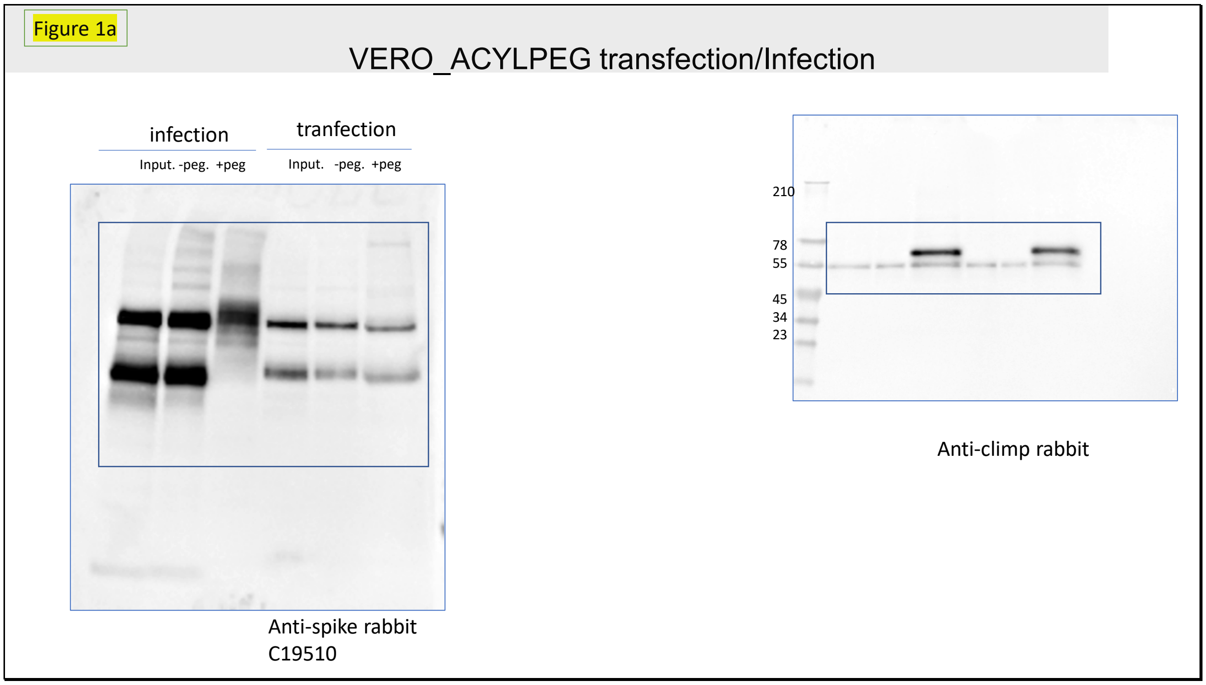

1b

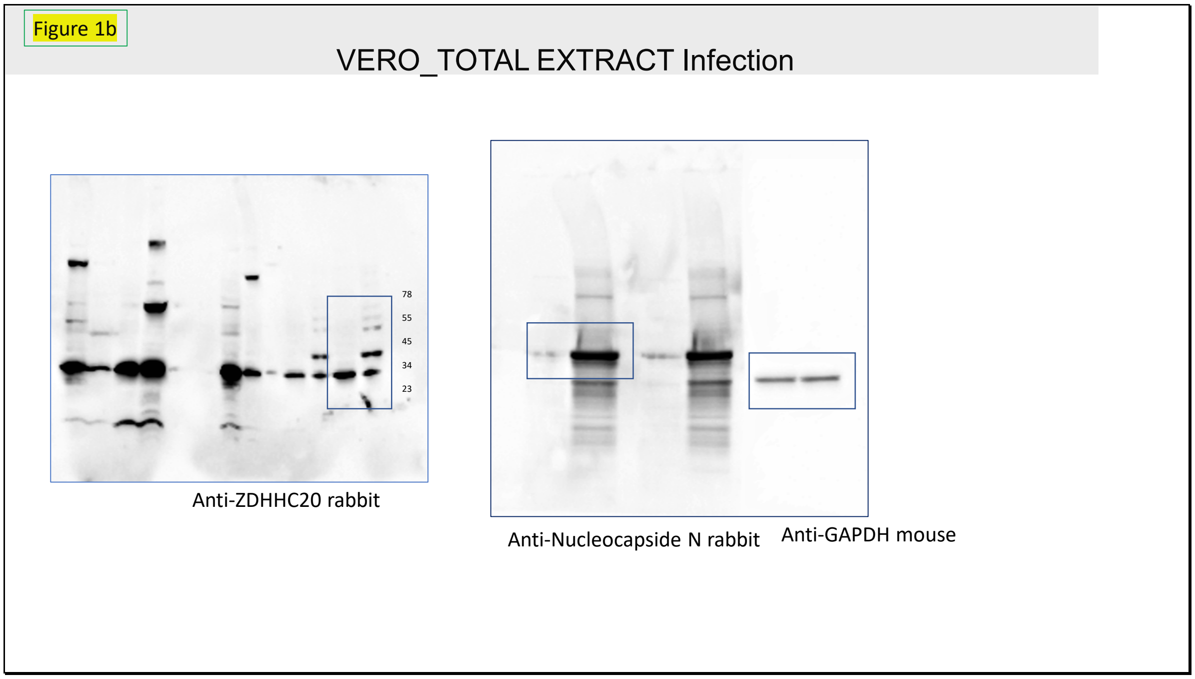

1c

Figure 1c

VERO_TOTAL EXTRACT Infection

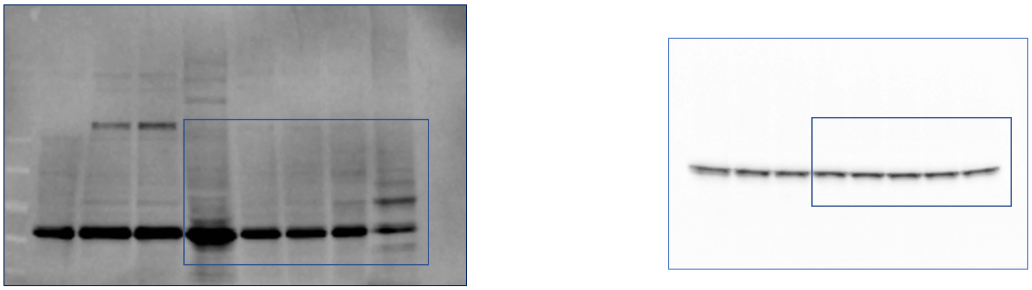

Anti-ZDHHC20 rabbit

Anti-GAPDH mouse

1e

Figure 1e

Human Airway_TOTAL EXTRACT Infection

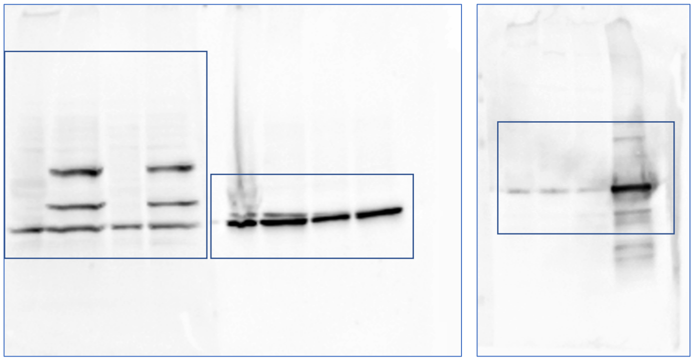

Anti-ZDHHC20 rabbit

Anti-GAPDH mouse

Anti-N Nucleocapside rabbit

1h

Figure 1H

VERO_TOTAL EXTRACT transfection

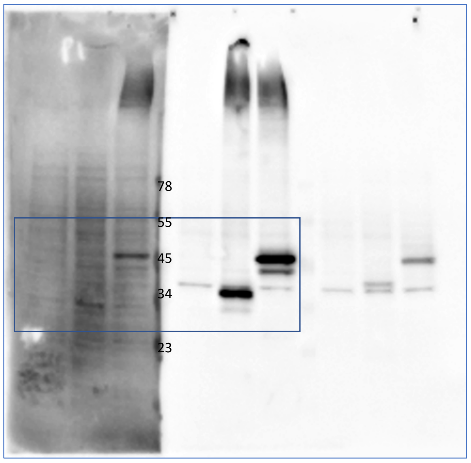

Anti-ZDHHC20-LONG rabbit   Anti-myc-zdhhc20 mouse

1i

Figure 1i

VERO_total cell extract_infection

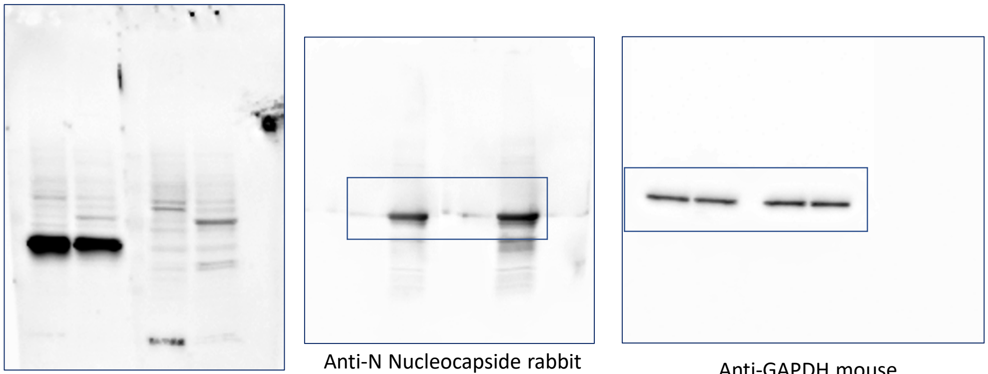

Anti-ZDHHC20 all rabbit   Anti-ZDHHC20 LONG rabbit   Anti-N Nucleocapside rabbit   Anti-GAPDH mouse

1j

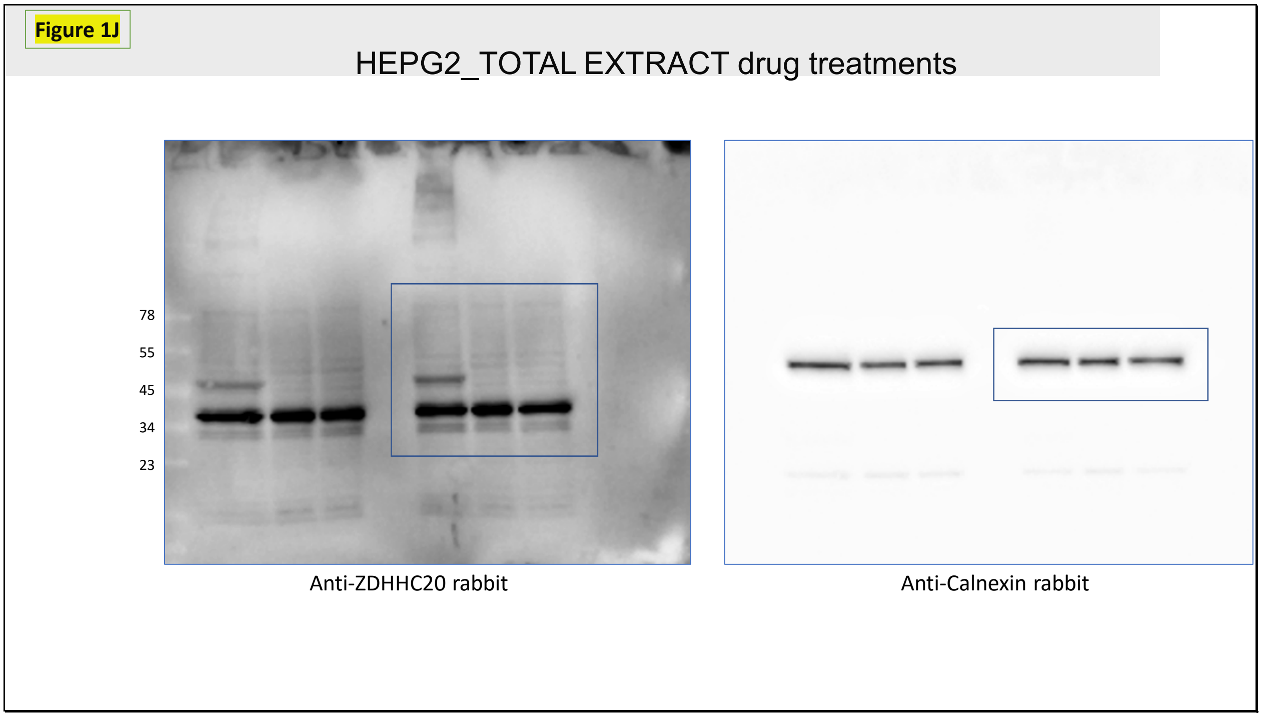
Figure 1J
HEPG2_TOTAL EXTRACT drug treatments
78
55
45
34
23
Anti-ZDHHC20 rabbit
Anti-Calnexin rabbit

1k

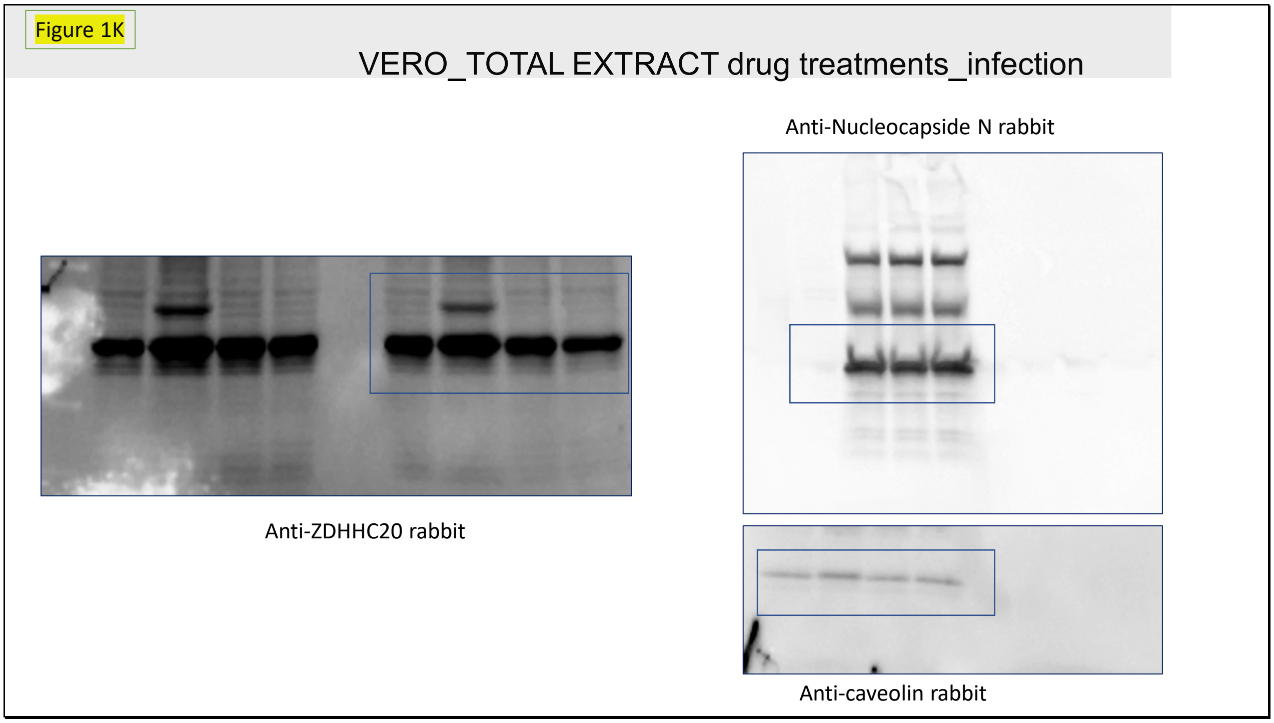
Figure 1K
VERO_TOTAL EXTRACT drug treatments_infection
Anti-Nucleocapside N rabbit
Anti-ZDHHC20 rabbit
Anti-caveolin rabbit

Supplementary Figure 1

S1a

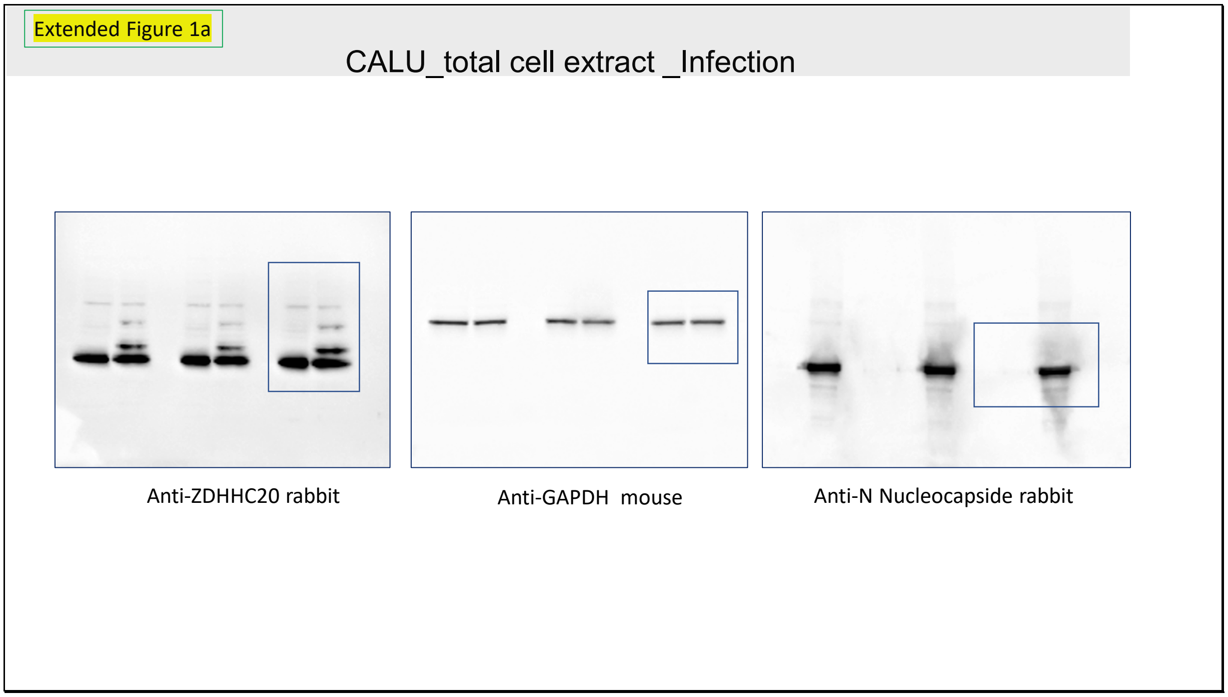

S1b

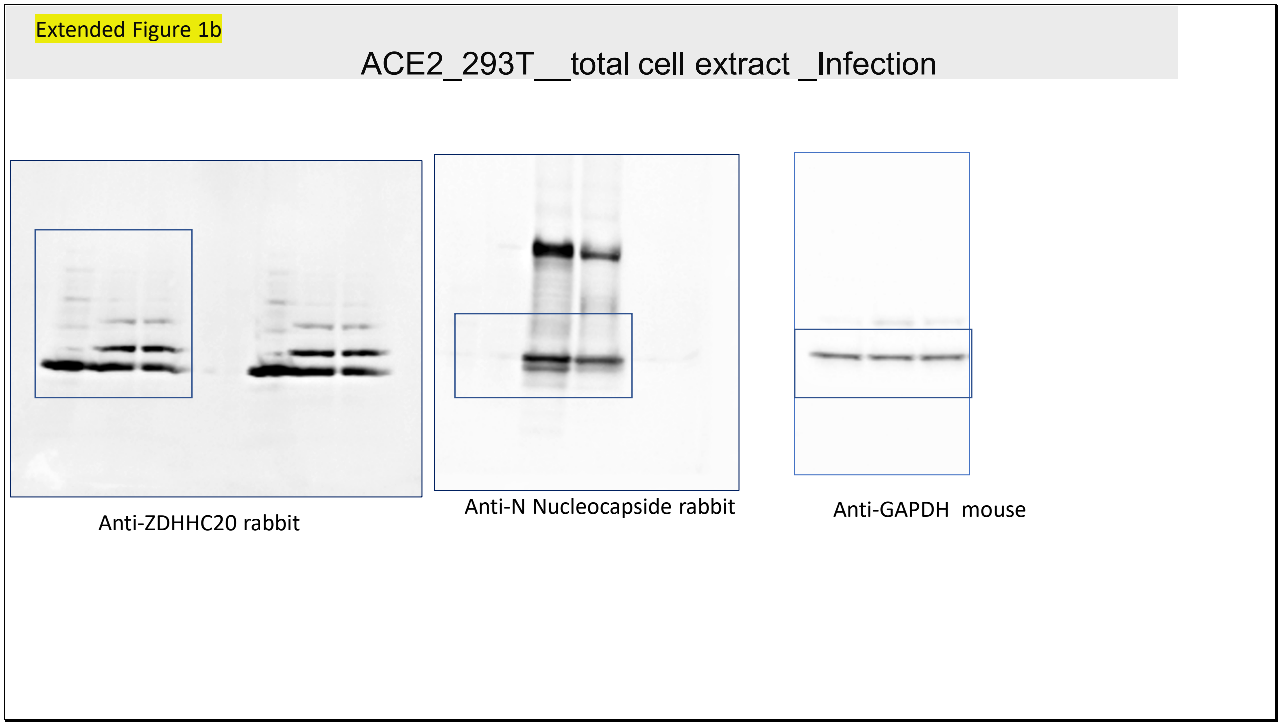

S1f

Extended Figure 1f

VERO_TOTAL EXTRACT Infection-transfection

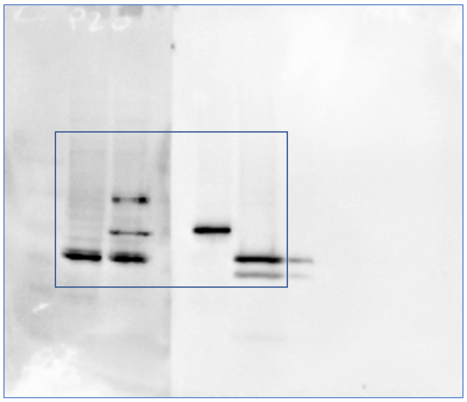

Anti-ZDHHC20 rabbit   Anti-myc-zdhhc20 mouse

S1h

Extended Figure 1h

HEPG2_TOTAL EXTRACT siRNA-transfection

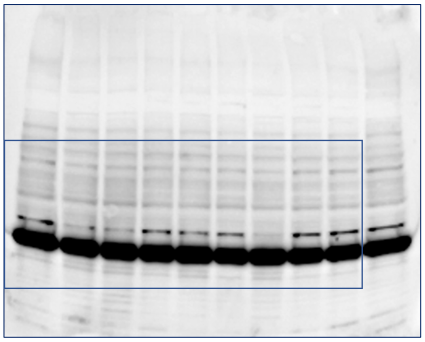

Anti-ZDHHC20 rabbit

S1j

Extended Figure 1j

Hela_cells_TOTAL EXTRACT transfection

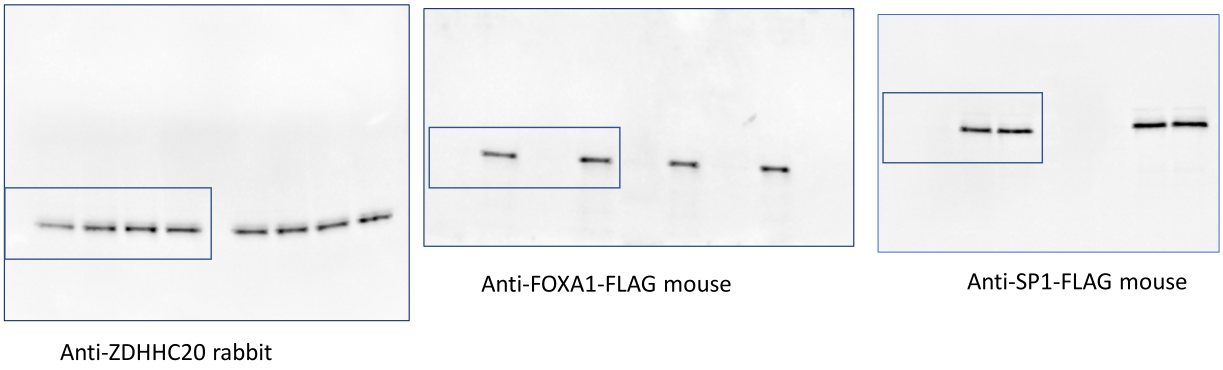

Figure 2

2a

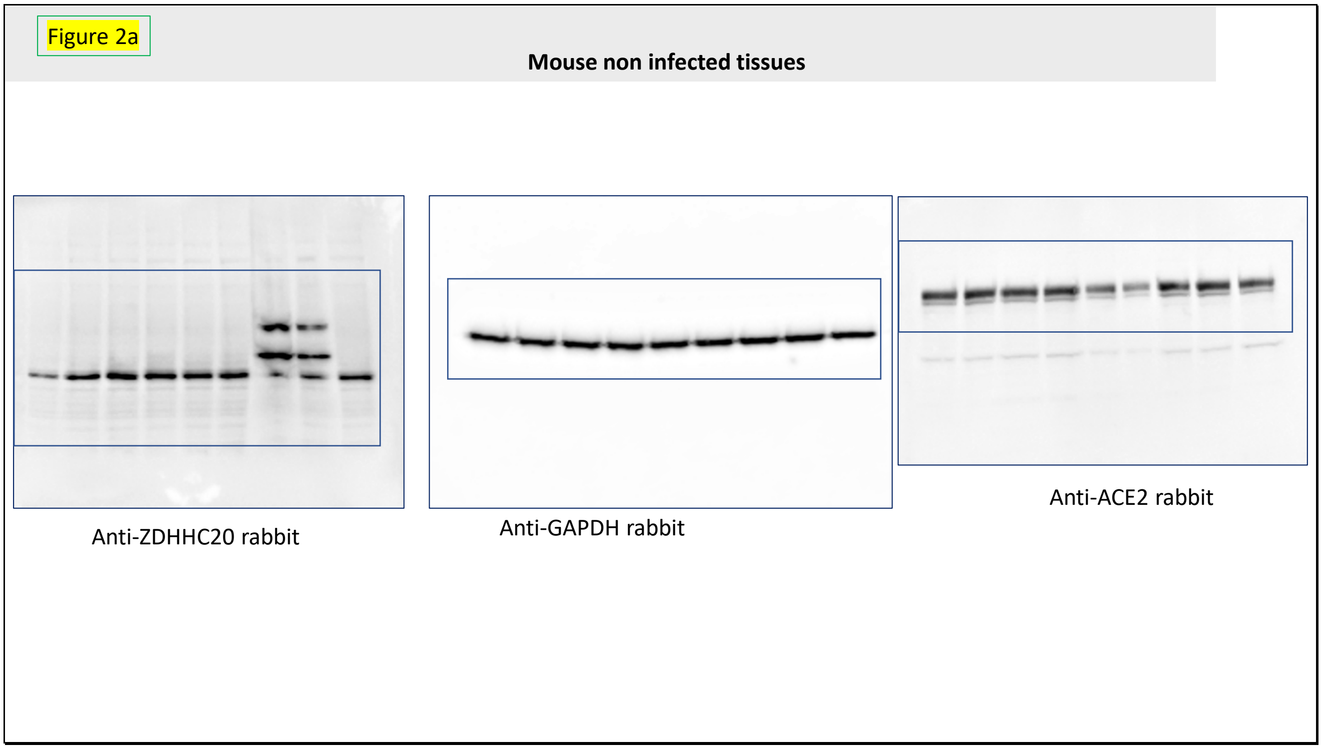

2e

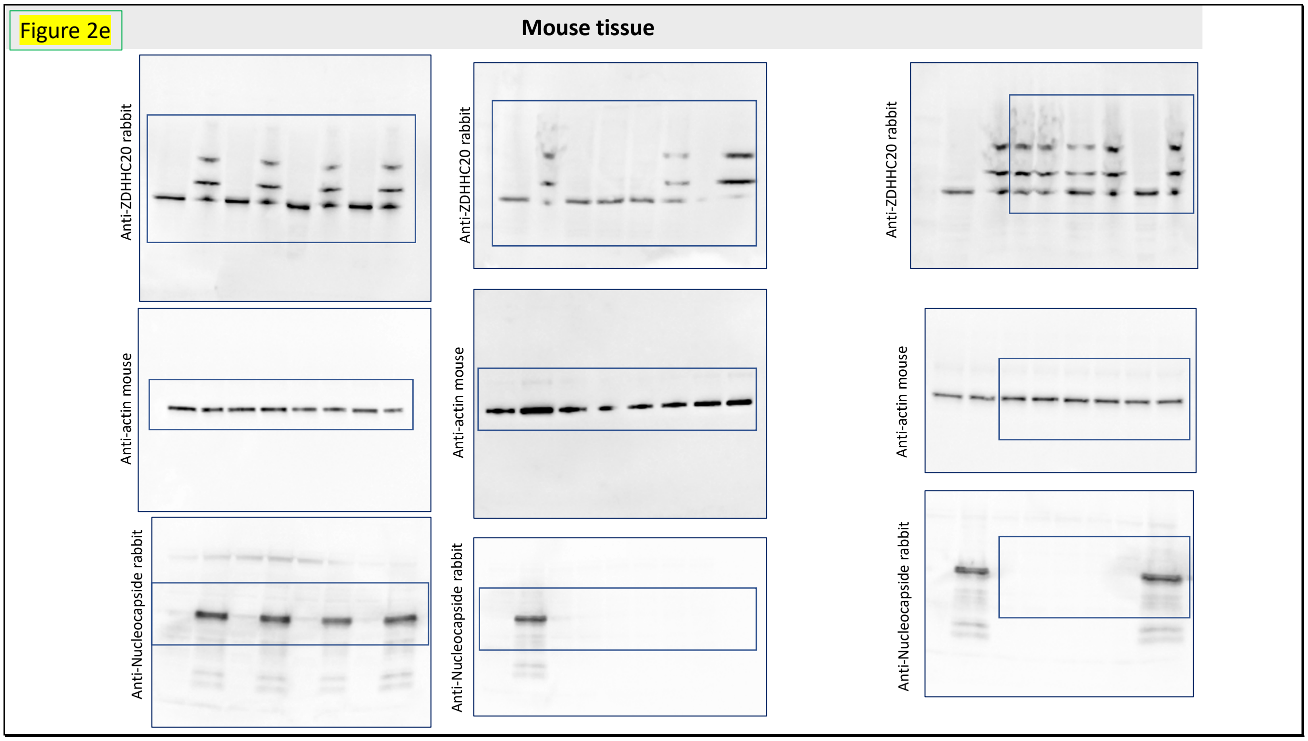

Figure 3

3b

Figure 3b

Colon

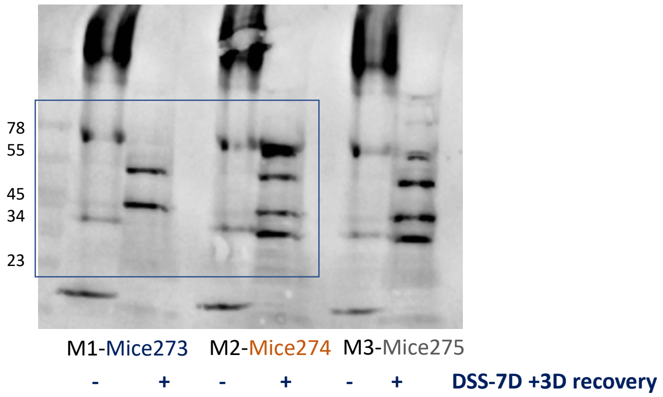

Anti-ZDHHC20 rabbit

3d

Figure 3d

Proaerolysin-Total cell extracts

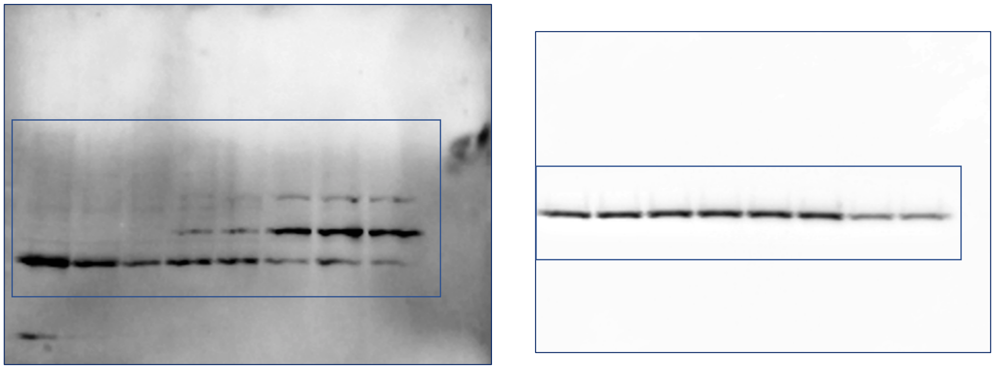

Anti-ZDHHC20 rabbit

Anti-GAPDH mouse

Supplementary Figure 3

S3f

Extended figure 3f

Continous Proaerolysin-Total cell extracts

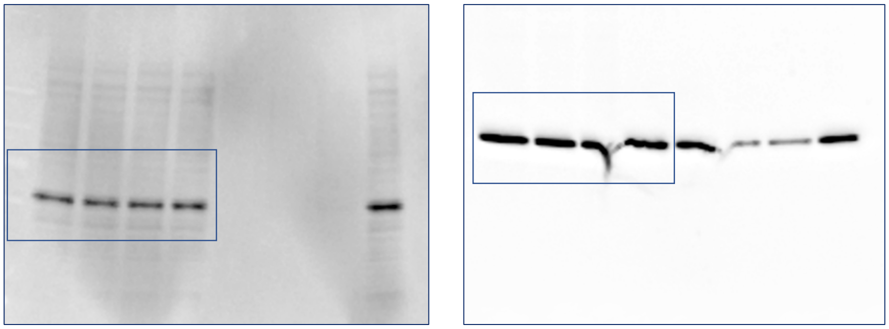

Anti-ZDHHC20 rabbit

Anti-GAPDH mouse

S3g

Extended figure 3g

Proaerolysin HELA-Total cell extracts

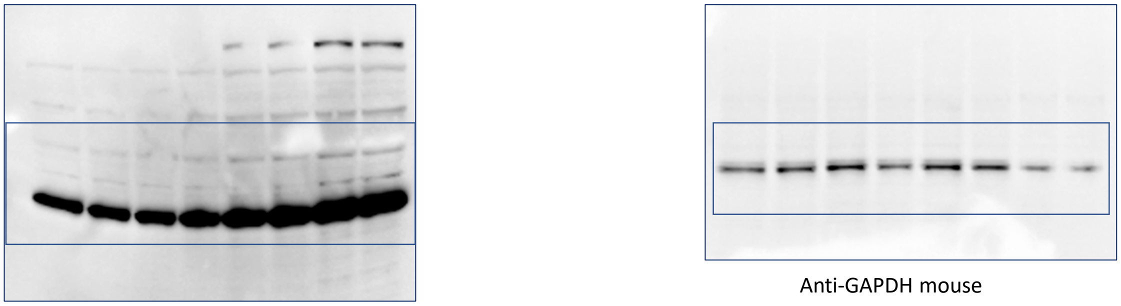

Anti-ZDHHC20 rabbit

Anti-GAPDH mouse

S3h

Extended figure 3h

Proaerolysin VERO E6-Total cell extracts

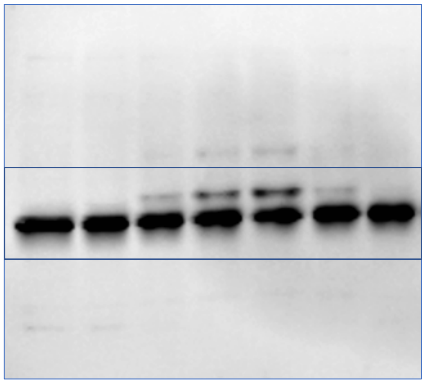

Anti-ZDHHC20 rabbit

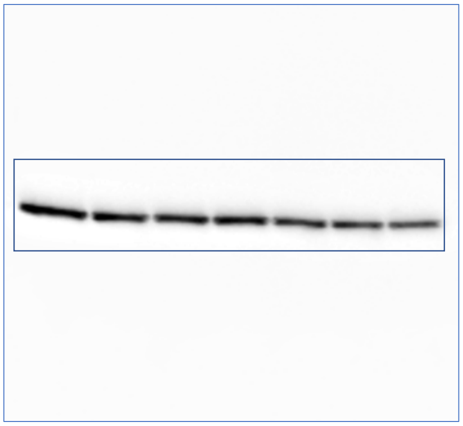

Anti-GAPDH mouse

Figure 4

4a

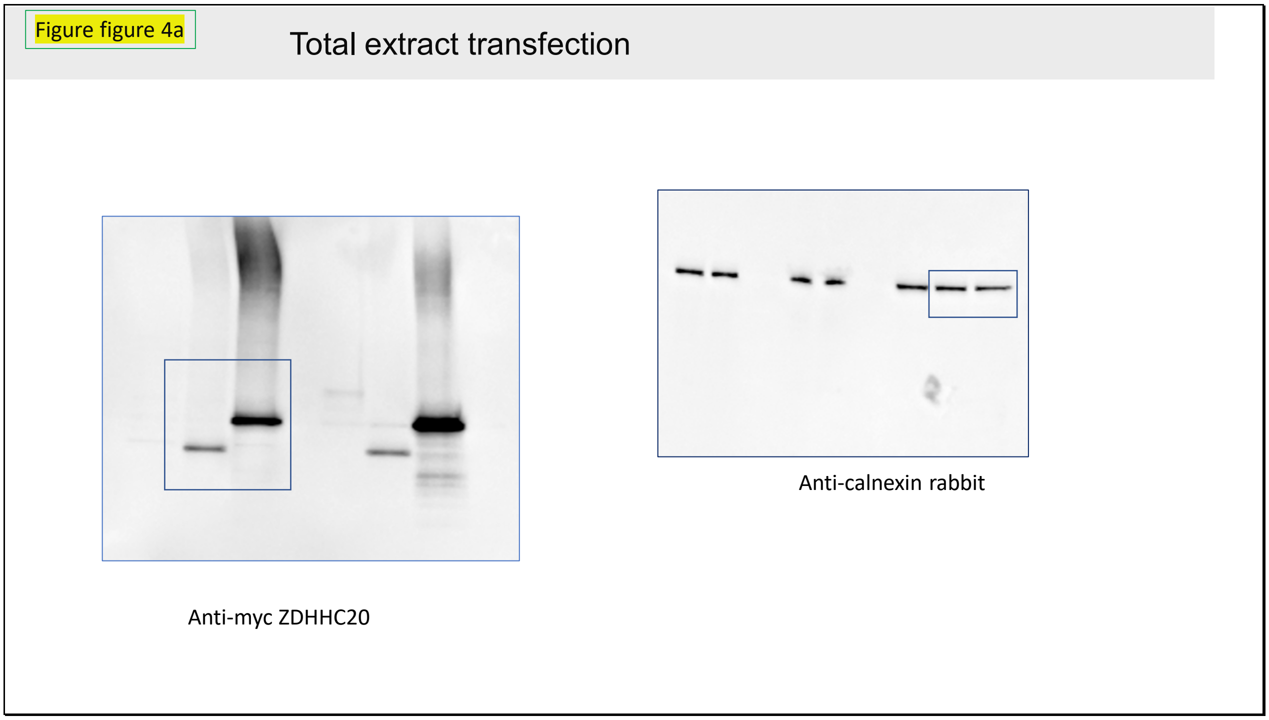

4l

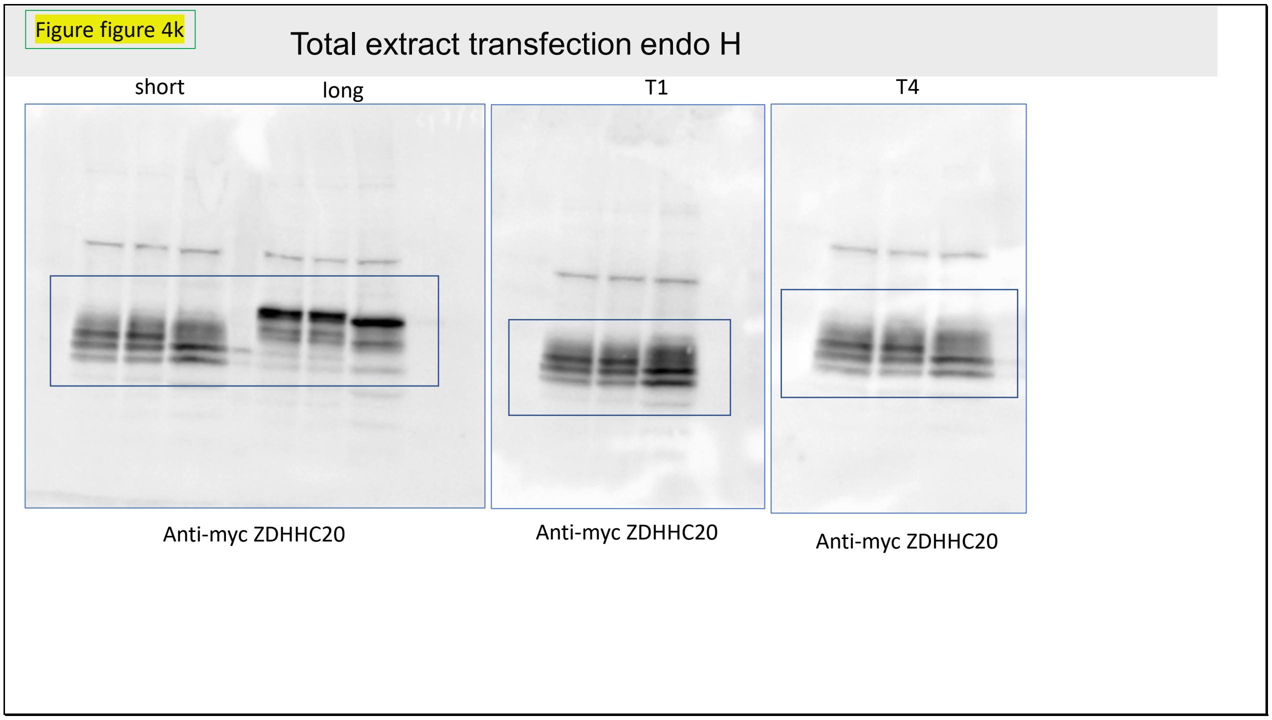

4l (continued)

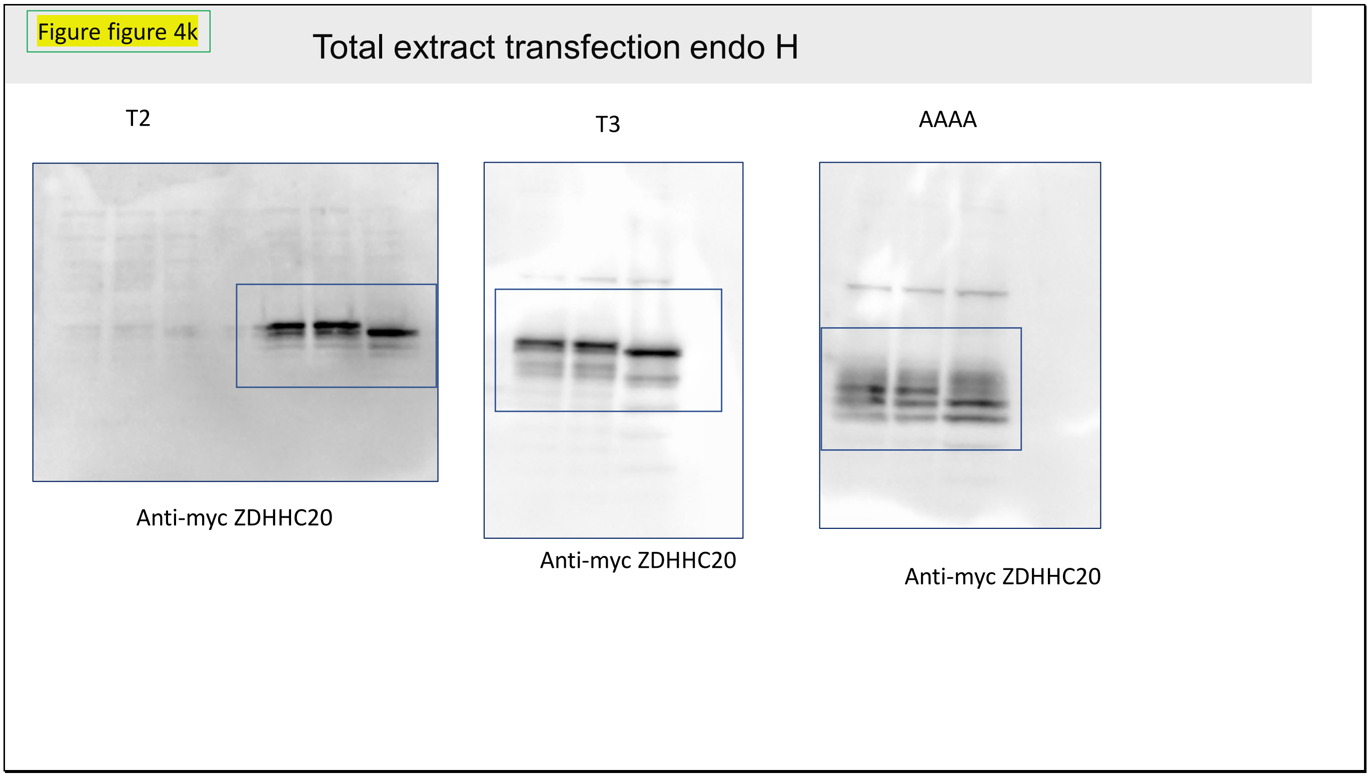

Figure 5

5f

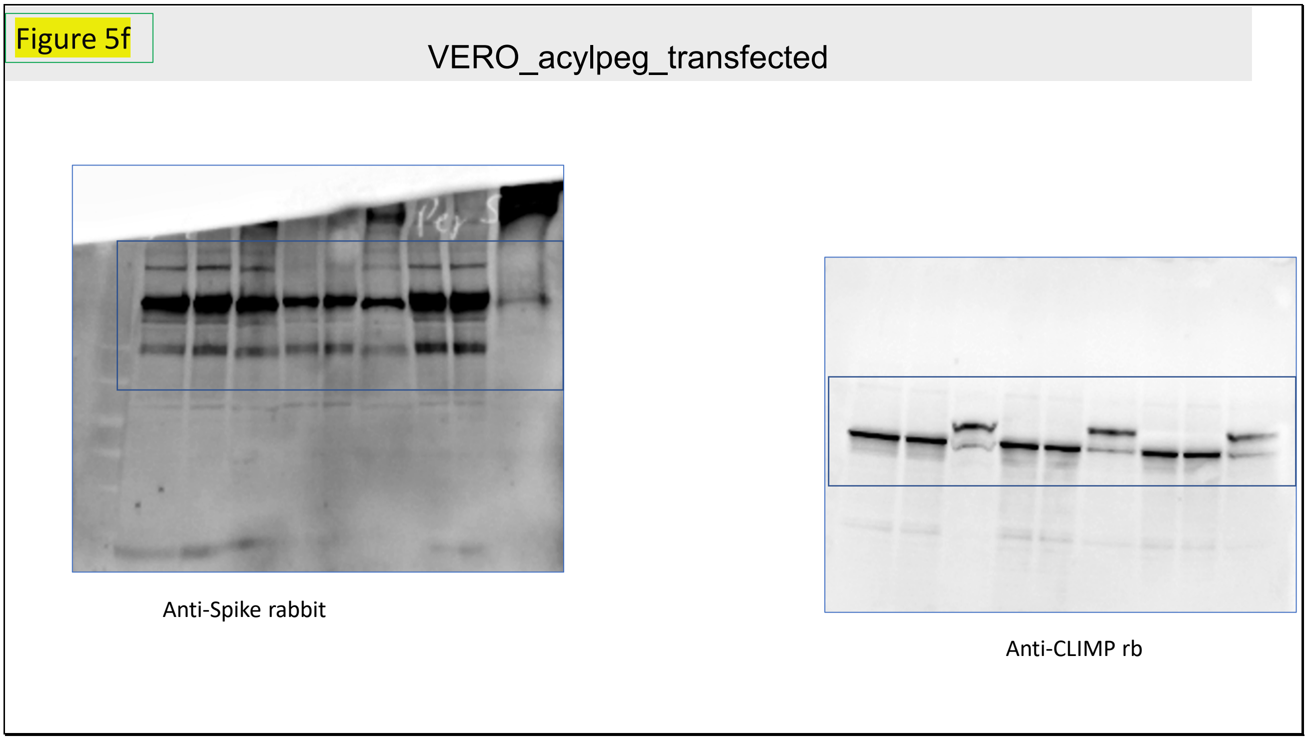

5g

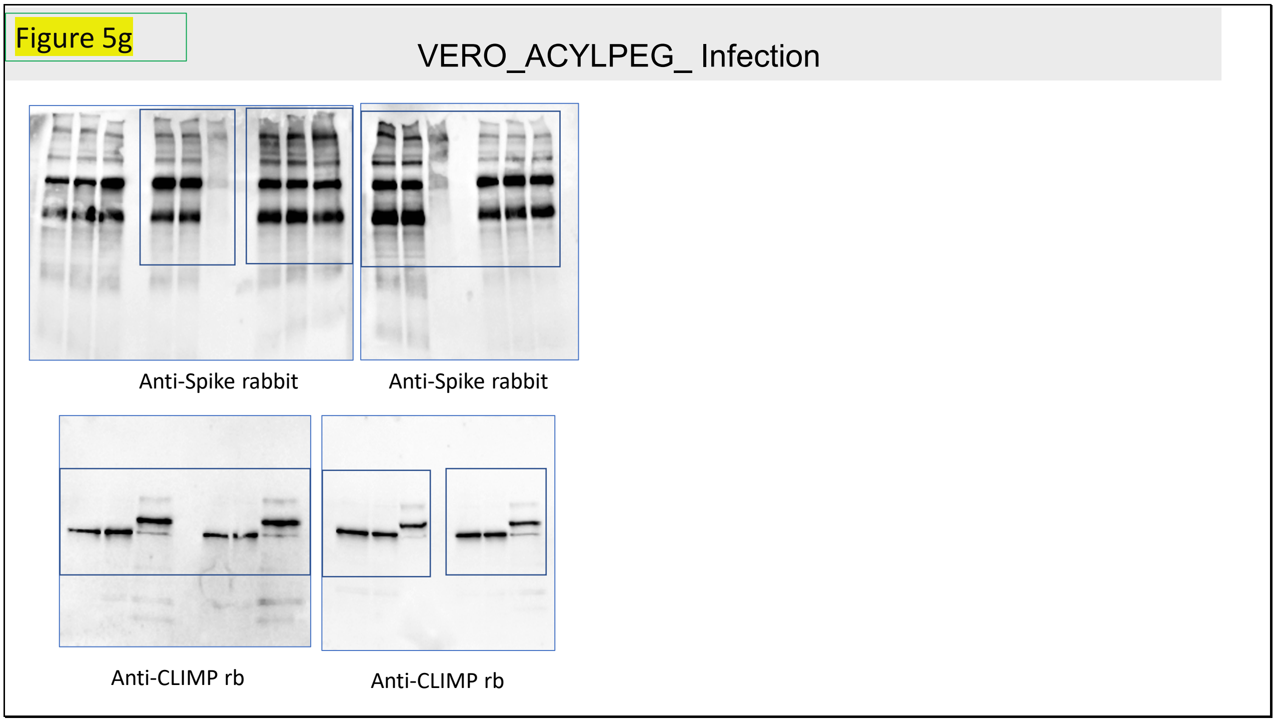

Supplementary Figure 5
S5b

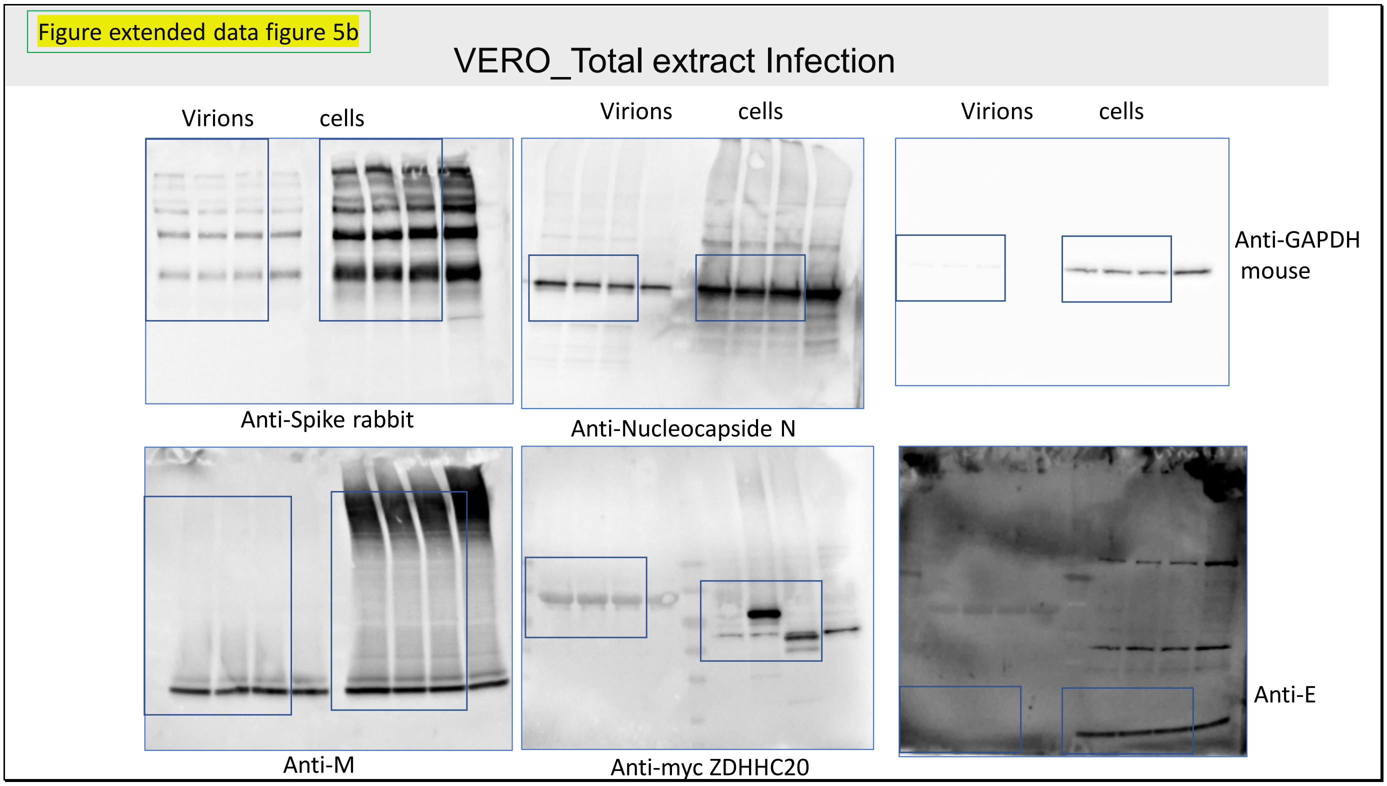

Supplement: Supplementary file 1 — Supplementary Information [file 41467_2023_43027_MOESM1_ESM.pdf]
