# Supplementary material for: SARS-CoV-2 hijacks a cell damage response, which induces transcription of a more efficient Spike S-acyltransferase
Source: Nat Commun. 2023 Nov 11;14:7302. doi: 10.1038/s41467-023-43027-2 (PMC10640587; doi:10.1038/s41467-023-43027-2)
Supplement: Supplementary file 3 — Description of Additional Supplementary Files [file 41467_2023_43027_MOESM3_ESM.docx]

**Description of additional supplementary files**

**Title: Supplementary Movie 1.**

**Description:** Time-lapse confocal microscopy (7 frames per second) of Vero E6 cells co-expressing (24 h) the Golgi marker Scarlet-Giantin, ZDHHC20^Long^-RUSH-GFP reporter and an ER-resident hook. Synchronized trafficking was monitored upon D-biotin addition, after T_0_ (see also Fig 4k and Extended data Fig 4f). Scale bar 10 μm.

**Title: Supplementary Movie 2.**

**Description:** Time-lapse confocal microscopy (7 frames per second) of Vero E6 cells co-expressing (24 h) the Golgi marker Scarlet-Giantin, ZDHHC20short-RUSH-GFP reporter and an ER-resident hook. Synchronized trafficking was monitored upon D-biotin addition after T_0_ (see also Fig 4k and Extended data Fig 4f). Scale bar 10 μm.
