# Supplementary material for: SARS-CoV-2 hijacks a cell damage response, which induces transcription of a more efficient Spike S-acyltransferase
Source: Nat Commun. 2023 Nov 11;14:7302. doi: 10.1038/s41467-023-43027-2 (PMC10640587; doi:10.1038/s41467-023-43027-2)
Supplement: Supplementary file 6 — Reporting Summary [file 41467_2023_43027_MOESM6_ESM.pdf]

nature portfolio

Corresponding author(s): Gisou van der Goot & Francisco Sarmento Mesquita
NMICROBIOL-23051120-T

Last updated by author(s): May 12, 2023

Reporting Summary

Nature Portfolio wishes to improve the reproducibility of the work that we publish. This form provides structure for consistency and transparency in reporting. For further information on Nature Portfolio policies, see our Editorial Policies and the Editorial Policy Checklist.

Statistics

For all statistical analyses, confirm that the following items are present in the figure legend, table legend, main text, or Methods section.

n/a	Confirmed	
☐	☒	The exact sample size (*n*) for each experimental group/condition, given as a discrete number and unit of measurement
☐	☒	A statement on whether measurements were taken from distinct samples or whether the same sample was measured repeatedly
☐	☒	The statistical test(s) used AND whether they are one- or two-sided<br>*Only common tests should be described solely by name; describe more complex techniques in the Methods section.*
☐	☒	A description of all covariates tested
☐	☒	A description of any assumptions or corrections, such as tests of normality and adjustment for multiple comparisons
☐	☒	A full description of the statistical parameters including central tendency (e.g. means) or other basic estimates (e.g. regression coefficient) AND variation (e.g. standard deviation) or associated estimates of uncertainty (e.g. confidence intervals)
☐	☒	For null hypothesis testing, the test statistic (e.g. *F*, *t*, *r*) with confidence intervals, effect sizes, degrees of freedom and *P* value noted<br>*Give P values as exact values whenever suitable.*
☒	☐	For Bayesian analysis, information on the choice of priors and Markov chain Monte Carlo settings
☒	☐	For hierarchical and complex designs, identification of the appropriate level for tests and full reporting of outcomes
☒	☐	Estimates of effect sizes (e.g. Cohen's *d*, Pearson's *r*), indicating how they were calculated

Our web collection on statistics for biologists contains articles on many of the points above.

Software and code

Policy information about availability of computer code

Data collection

- Microscopy images were acquired:
Upright Olympus motorized BX 61 V with Olympus OlyVIA software (Fig 2,3 and extended data Fg 3); or
Inverted Zeiss AxioObserver Z1 with using ZEN 2009 (Fig 4 and extend data Fig 4 except for 4J and extended data 4f);
Visitron Spinning Disk CSU W1, Inverted Olympus IX 83 motorized, with VisiView. (Fig 4J and extended data 4f and supplementary movies)
- Microscopy images were processed using QuPath_v4 or Fiji v1.54d
- Western blot images collected with fusion solo 65 edge.
- Autoradiography signals were collected using Typhoon phosphoimager and quantified using the Typhoon Imager (ImageQuanTool, GE Healthcare)
- qPCR data was collected using 7900HT Fast QPCR Applied Biosystems with SDS 2.4 Software.
- Bioluminescence data was collected using HIDEX microplate reader , HIDEX software v1.3.0
- Statistical data was retrieved using Prism Graphpad v9

Data analysis

Code used for RNAscope quantification in Fig 2, 3 and Supplementary data fig 3

```
* = CODE DESCRIPTION =
 * This script will apply a Laplacian of Gaussian filtered followed by a local maxima detector in order to locate
 * RNAScope spots in fluorescent images. The script works with an arbitrary number of channels, provided as a list.
 *
 * == MATERIALS & METHODS ==
 * RNAScope spots are identified using a Laplacian of Gaussian[1] filter (LoG) followed by a 2D local maximum finder.
```

```
 * In summary, annotated regions are imported from QuPath into ImageJ[2] and run through a median filter (radius=1.5 px)
 * in order to remove Poisson noise.
 * The diffraction-limited spots are then enhanced using a LoG filter (sigma=1.0 px). The resulting image is run through
 * ImageJ's local maximum finder algorithm with a channel-dependent tolerance value[3].
 * The local maxima points are then reimported into QuPath for each channel.
 *
 * REFERENCES
 * ----------
 * [1] https://imagescience.org/meijering/software/featurej/laplacian/
 * [2] Schneider, C. A., Rasband, W. S., & Eliceiri, K. W. (2012). NIH Image to ImageJ: 25 years of image analysis. Nature Methods, 9(7), 671–675. doi:10.1038/nmeth.2089
 * [3] https://github.com/imagej/ImageJ/blob/master/ij/plugin/filter/MaximumFinder.java
 *
 * == INPUTS ==
 * A single or multichannel fluorescence image with annotations. This script will run on all annotations
 * The user should specify the channel names to use, as well as the prominence values for each channel.
 * Higher prominence values will find fewer spots, as it represents how much of a difference the peaks should have with their
 * local background.
 *
 * == OUTPUTS ==
 * After running the code, mRNA spots will be shown Points, in the color of the channel.
 * Two new measurements "RNAScope CHANNEL Spots" and RNASctop CHANNEL Density are also appended to each annotation
 *
 * = DEPENDENCIES =
 * This script makes use of the ImageScience library at https://imagescience.org/meijering/software/imagescience/
 *
 * = INSTALLATION =
 * You must downloaAll code details are available in GitHub (https://github.com/upvdg/rnascope-qupath)d 'imagescience.jar' and place it into your QuPath extensions directory
 * https://imagescience.org/meijering/software/imagescience/
 *
 * = AUTHOR INFORMATION =
 * Code written by Olivier Burri, EPFL - SV - PTECH - BIOP
 * for Lucie Bracq, Van Der Goot Lab
 * Last update: 20230314
 *
 * = COPYRIGHT =
 * © All rights reserved. ECOLE POLYTECHNIQUE FEDERALE DE LAUSANNE, Switzerland, BioImaging and Optics Platform (BIOP), 2023
All code details are available in GitHub (https://github.com/upvdg/rnascope-qupath)
```

For manuscripts utilizing custom algorithms or software that are central to the research but not yet described in published literature, software must be made available to editors and reviewers. We strongly encourage code deposition in a community repository (e.g. GitHub). See the Nature Portfolio guidelines for submitting code & software for further information.

Data

Policy information about availability of data

All manuscripts must include a data availability statement. This statement should provide the following information, where applicable:

- Accession codes, unique identifiers, or web links for publicly available datasets
- A description of any restrictions on data availability
- For clinical datasets or third party data, please ensure that the statement adheres to our policy

All Raw data are provided has a Source data file. All Code details are provided. All data supporting the findings of this study are available within the paper, the Supplementary Information and Supplementary Data Source files. Source data are provided as a Source Data file. All data regarding the human zdhhc20 locus was obtained from the Genome browser on Human (GRCh37/hg19) (version hg19); Transcript data obtained from GENCODE Genes track (V4 0lift37) https://genome.ucsc.edu/cgi-bin/hgTrackUi?g=knownGene ; Chromatin State segmentation obtained from ENCODE https://genome.ucsc.edu/cgi-bin/hgTrackUi?db=hg18&g=wgEncodeBroadHmm. All data from murine zdhhc20 locus was obtained from Mouse GRCm39/mm39 from UCSC genome browser https://genome.ucsc.edu.

Research involving human participants, their data, or biological material

Policy information about studies with human participants or human data. See also policy information about sex, gender (identity/presentation), and sexual orientation and race, ethnicity and racism.

Reporting on sex and gender	N/A
Reporting on race, ethnicity, or other socially relevant groupings	N/A

Population characteristics	N/A
Recruitment	N/A
Ethics oversight	NA

Note that full information on the approval of the study protocol must also be provided in the manuscript.

Field-specific reporting

Please select the one below that is the best fit for your research. If you are not sure, read the appropriate sections before making your selection.

[x] Life sciences [ ] Behavioural & social sciences [ ] Ecological, evolutionary & environmental sciences

For a reference copy of the document with all sections, see nature.com/documents/nr-reporting-summary-flat.pdf

Life sciences study design

All studies must disclose on these points even when the disclosure is negative.

Sample size	All sample sizes and correspondent statistical comparisons are indicated in figure legends or methods sections. For microscopy qualitative quantifications the<br>number of analyzed cells per set is indicated. For all experiments we chose to analyse three or more biological independent samples/assays, and the N is detailed for each experiment. Due to the large sample size for the high-trough put image quantifications, two independent experiments with 4 wells, 9 images per well were performed. Total number of cells<br>are indicated
Data exclusions	no data was excluded, and all experiment results are detailed
Replication	For all experiments we chose to analyses three or more biological independent assays.
Randomization	non applicable to this study because human bias were not present in the presented measurements
Blinding	The image analysis was either done automatedly or in unmarked microscopic slides. The remaining experiments were not subjected to bias

Reporting for specific materials, systems and methods

We require information from authors about some types of materials, experimental systems and methods used in many studies. Here, indicate whether each material, system or method listed is relevant to your study. If you are not sure if a list item applies to your research, read the appropriate section before selecting a response.

Materials & experimental systems

n/a	Involved in the study	
[ ]	[x]	Antibodies
[ ]	[x]	Eukaryotic cell lines
[x]	[ ]	Palaeontology and archaeology
[ ]	[x]	Animals and other organisms
[x]	[ ]	Clinical data
[x]	[ ]	Dual use research of concern
[x]	[ ]	Plants

Methods

n/a	Involved in the study	
[x]	[ ]	ChIP-seq
[x]	[ ]	Flow cytometry
[x]	[ ]	MRI-based neuroimaging

Antibodies

Antibodies used	ACE-2 (Abcam: ab15348; RRID: AB_301861; rabbit: 1:2000 dilution).<br>Actin (Millipore: MAB1501: RRID: AB_2223041; mouse: 1:4000 dilution).<br>Calnexin (Millipore: MAB3126: RRID: 2069152; mouse: 1:2000 dilution).<br>Climp63/CKAP4 (Bethyl Laboratories: A302-257A; RRID: AB_1731083; rabbit: 1: 2000 dilution).<br>GAPDH (Thermofisher: 398600; RRID: AB_2533438; mouse: 1:4000 dilution).<br>Giantin (Abcam: ab37266; RRID: AB_880195; rabbit: 1:200 dilution).<br>GOLPH3 (Abcam: ab98023; RRID:AB_10860828; rabbit : 1:200 dilution).<br>GM130 (BD:610823; RRID: AB_3998141; mouse: 1:200 dilution).<br>HA (Roche: 11867423001; RRID: AB_390918; rat: 1:500 dilution).<br>Keratin20 KRT20 (Cell Signalling: 13063; RRID: AB_2798106; rabbit: 1:600 dilution).<br>myc (Sigma: M4439; RRID: AB_439694; mouse: 1:2000 dilution).

Nucleocapside N SARS-CoV-2 (Genetex: GTX135357; RRID: AB_2868464; rabbit 1:2000 dilution).
SARS-CoV-1/2 E and M antibodies are gift from Machamer lab.
OLFM4 (Cell Signalling: 39141; RRID:AB_2650511; rabbit: 1:250 dilution).
Spike SARS-CoV-2 (Lifespan: LS-C19510; RRID: AB_840148; rabbit: 1:2000 dilution).
ZDHHC20 ALL (Sigma: SAB4501054; RRID: AB_10744838; rabbit: 1: 2000 dilution).
Mouse-HRP (GEHealthcare: NA931V; RRID: AB_772210; mouse: 1: 3000 dilution).
Rabbit-HRP (GEHealthcare: NA934V; RRID: AB_772206; rabbit: 1: 3000 dilution).
Mouse-Alexa488 (ThermoFisher Scientific: A-11029; RRID: AB_2534088; 1:800 dilution).
Mouse-Alexa568 (ThermoFisher Scientific: A-11037; RRID: AB_2534013; 1:800 dilution).
Rabbit-Alexa488 (ThermoFisher Scientific: A-21206; RRID: AB_2535792; 1:800 dilution).
Rabbit-Alexa568 (ThermoFisher Scientific: A-11042; RRID: AB_2534017; 1:800 dilution).
DAPI (ThermoFisher Scientific: D1306; RRID: AB_2629482; 1:5000 dilution).
Hoechst (Sigma: 94403; 1:5000 dilution).

Polyclonal Antibodies production against Long ZDHHC20 Long
The 2 following peptides were used in combination to produce polyclonal antibodies in 2 different rabbits, 2 serum were pooled and were immunopurified against the 2 peptides (Peptide 1: C-EAGELDQQPPGASES-coNH2- Peptide 2: C-LPRSPERWDAGLGSRQR-coNH2).
ACE-2 (Abcam: ab15348; RRID: AB_301861; rabbit: 1:2000 dilution).
Actin (Millipore: MAB1501: RRID: AB_2223041; mouse: 1:4000 dilution).
Calnexin (Millipore: MAB3126: RRID: 2069152; mouse: 1:2000 dilution).
Climp63/CKAP4 (Bethyl Laboratories: A302-257A; RRID: AB_1731083; rabbit: 1: 2000 dilution).
Flag (Sigma: F3165; RRID: AB_259529; mouse : 1:2000).
GAPDH (Thermofisher: 398600; RRID: AB_2533438; mouse: 1:4000 dilution).
Giantin (Abcam: ab37266; RRID: AB_880195; rabbit: 1:200 dilution).
GOLPH3 (Abcam: ab98023; RRID:AB_10860828; rabbit : 1:200 dilution).
GM130 (BD:610823; RRID: AB_3998141; mouse: 1:200 dilution).
HA (Roche: 11867423001; RRID: AB_390918; rat: 1:500 dilution).
Keratin20 KRT20 (Cell Signalling: 13063; RRID: AB_2798106; rabbit: 1:600 dilution).
myc (Sigma: M4439; RRID: AB_439694; mouse: 1:2000 dilution).
Nucleocapside N SARS-CoV-2 (Genetex: GTX135357; RRID: AB_2868464; rabbit 1:2000 dilution).
SARS-CoV-1/2 E and M antibodies are gift from Machamer lab.
OLFM4 (Cell Signalling: 39141; RRID:AB_2650511; rabbit: 1:250 dilution).
Spike SARS-CoV-2 (Lifespan: LS-C19510; RRID: AB_840148; rabbit: 1:2000 dilution).
ZDHHC20 ALL (Sigma: SAB4501054; RRID: AB_10744838; rabbit: 1: 2000 dilution).
Mouse-HRP (GEHealthcare: NA931V; RRID: AB_772210; mouse: 1: 3000 dilution).
Rabbit-HRP (GEHealthcare: NA934V; RRID: AB_772206; rabbit: 1: 3000 dilution).
Mouse-Alexa488 (ThermoFisher Scientific: A-11029; RRID: AB_2534088; 1:800 dilution).
Mouse-Alexa568 (ThermoFisher Scientific: A-11037; RRID: AB_2534013; 1:800 dilution).
Rabbit-Alexa488 (ThermoFisher Scientific: A-21206; RRID: AB_2535792; 1:800 dilution).
Rabbit-Alexa568 (ThermoFisher Scientific: A-11042; RRID: AB_2534017; 1:800 dilution).
DAPI (ThermoFisher Scientific: D1306; RRID: AB_2629482; 1:5000 dilution).
Hoechst (Sigma: 94403; 1:5000 dilution).

Polyclonal Antibodies production against Long ZDHHC20 Long
The 2 following peptides were used in combination to produce polyclonal antibodies in 2 different rabbits, 2 serum were pooled and were immunopurified against the 2 peptides (Peptide 1: C-EAGELDQQPPGASES-coNH2- Peptide 2: C-LPRSPERWDAGLGSRQR-coNH2).

Validation

- ACE-2 (Abcam: ab15348; RRID: AB_301861; rabbit: 1:2000 dilution). Detailed validation from manufacturer's page: Use a concentration of 1 - 2 µg/ml. Detects a band of approximately 90 kDa (predicted molecular weight: 97 kDa).
- Actin (Millipore: MAB1501: RRID: AB_2223041; mouse: 1:4000 dilution). Detailed validation from manufacturer's page: Immunoblots use at 1:100-1:1,000 (Otey, 1987):On muscle homogenates subject to SDS-PAGE, reacts relatively uniformly with a 43 kD protein present in skeletal, cardiac, gizzard and aorta tissues. Appears to react with all isoforms of actin found in these preparations and shows a strong reaction with the alpha-actin found in skeletal, cardiac, and arterial muscle.
- Calnexin (Millipore: MAB3126: RRID: 2069152; mouse: 1:2000 dilution). Detailed validation from manufacturer's page: Western Blotting Analysis: A representative lot detected Calnexin in Western Blotting applications (Hochstenbach, F., et. al. (1992). Proc Natl Acad Sci USA. 89(10):4734-8). Immunocytochemistry Analysis: A 1:250 dilution from a representative lot detected Calnexin in HeLa, A431 and Huvec cell lines.
- Climp63/CKAP4 (Bethyl Laboratories: A302-257A; RRID: AB_1731083; rabbit: 1: 2000 dilution). Detailed validation from manufacturer's page: Western Blot (WB) 1:2,000 - 1:10,000
- GAPDH (Thermofisher: 398600; RRID: AB_2533438; mouse: 1:4000 dilution). Detailed validation from manufacturer's page: Western Blot (WB) 0.5-2 µg/mL
- Giantin (Abcam: ab37266; RRID: AB_880195; rabbit: 1:200 dilution). Detailed validation from manufacturer's page: IF Use a concentration of 5 - 10 µg/ml.
- GOLPH3 (Abcam: ab98023; RRID:AB_10860828; rabbit : 1:200 dilution). Detailed validation from manufacturer's page: Use a concentration of 5 µg/ml
- GM130 (BD:610823; RRID: AB_3998141; mouse: 1:200 dilution). Detailed validation from manufacturer's page: Immunofluorescence (Tested During Development)
- HA (Roche: 11867423001; RRID: AB_390918; rat: 1:500 dilution). Detailed validation from manufacturer's page: working concentration of conjugate depends on application and substrate. The following concentrations should be taken as a guideline: ELISA:

for detection 100 ng/ml; for coating 1 to 5 µg/ml, Immunoprecipitation: 0.5 to 5 µg/ml, Western blot: 50 to 200 ng/ml
- Keratin20 KRT20 (Cell Signalling: 13063; RRID: AB_2798106; rabbit: 1:600 dilution). Detailed validation from manufacturer's page: Immunofluorescence (Immunocytochemistry) 1:200 - 1:800
- Myc (Sigma: M4439; RRID: AB_439694; mouse: 1:2000 dilution). Detailed validation from manufacturer's page: Immunocytochemistry (ICC/IF) - 1:100-1:500
- Nucleocapside N SARS-CoV-2 (Genetex: GTX135357; RRID: AB_2868464; rabbit 1:2000 dilution). Detailed validation from manufacturer's page: ICC/IF-1:100-1:1000
- SARS-CoV-1/2 E and M antibodies are gift from Machamer lab. validated in: Cohen, J. R., Lin, L. D. & Machamer, C. E. Identification of a Golgi Complex-Targeting Signal in the Cytoplasmic Tail of the Severe Acute Respiratory Syndrome Coronavirus Envelope Protein. Journal of Virology 85, 5794–5803 (2011). AND Mesquita, F. S. et al. S-acylation controls SARS-CoV-2 membrane lipid organization and enhances infectivity. Developmental Cell 56, 2790-2807.e8 (2021).
- OLFM4 (Cell Signalling: 39141; RRID:AB_2650511; rabbit: 1:250 dilution). Detailed validation from manufacturer's page: Immunofluorescence (Immunocytochemistry) – 1:100 to 1:300 – Citation - IFNγ-Stat1 axis drives aging-associated loss of intestinal tissue homeostasis and regeneration. In Nature Communications on 30 September 2023 by Omrani, O., Krepelova, A., et all Applications: IHC-IF - Reactivity: Mus musculus (House mouse)
- Spike SARS-CoV-2 (Lifespan: LS-C19510; RRID: AB_840148; rabbit: 1:2000 dilution). Mesquita, F. S. et al. S-acylation controls SARS-CoV-2 membrane lipid organization and enhances infectivity. Developmental Cell 56, 2790-2807.e8 (2021).
- ZDHHC20 ALL (Sigma: SAB4501054; RRID: AB_10744838; rabbit: 1: 2000 dilution). Detailed validation from manufacturer's page: western blot: 1:500-1:1000
- Secondary antibodies – All secondary antibodies have been validated in several published papers – The detailed validation of different papers is available from manufacturer's page:
- Mouse-HRP (GEHealthcare: NA931V; RRID: AB_772210; mouse: 1: 3000 dilution).
- Rabbit-HRP (GEHealthcare: NA934V; RRID: AB_772206; rabbit: 1: 3000 dilution).
- Mouse-Alexa488 (ThermoFisher Scientific: A-11029; RRID: AB_2534088; 1:800 dilution).
- Mouse-Alexa568 (ThermoFisher Scientific: A-11037; RRID: AB_2534013; 1:800 dilution).
- Rabbit-Alexa488 (ThermoFisher Scientific: A-21206; RRID: AB_2535792; 1:800 dilution).
- Rabbit-Alexa568 (ThermoFisher Scientific: A-11042; RRID: AB_2534017; 1:800 dilution).

- DAPI (ThermoFisher Scientific: D1306; RRID: AB_2629482; 1:5000 dilution). Detailed validation from manufacturer's page: dilute the DAPI stock solution to 300 nM in PBS. Add approx¬¬i¬mately 300 µL of this dilute DAPI staining solution to the coverslip preparation,
- Hoechst (Sigma: 94403; 1:5000 dilution). Detailed validation from manufacturer's protocol page: Hoechst 3358 (94403) counterstaining (1:500 dilution in PBS) can be added together with the secondary antibody to counterstain the cell nuclei (150 µl/well).

Eukaryotic cell lines

Policy information about cell lines and Sex and Gender in Research

Cell line source(s)	Vero E6 (ATCC: CVCL_0574), HEPG2 (ATCC: HB_8065), Calu-3 (ATCC: HTB_55), HELA (ATCC: CVCL_0030). HEK293TphACE2-TMPRSS2 cells were kindly provided by Priscilla Turrelly from Didier Trono Lab - validated/published in: Fenwick, C. et al. Broadly potent anti-SARS-CoV-2 antibody shares 93% of epitope with ACE2 and provides full protection in monkeys. Journal of Infection (2023) doi:10.1016/j.jinf.2023.10.008.and Fenwick, C. et al. Patient-derived monoclonal antibody neutralizes SARS-CoV-2 Omicron variants and confers full protection in monkeys. Nat Microbiol 7, 1376–1389 (2022).<br>Parental Vero E6 (ATCC: CVCL_0574), cell lines were used to engineer KOZDHHC20, KOZDHHC20/9 cells Vero E6. Cells were validated by genotyping and western blot in published study
Authentication	All parental cell lines were commercially acquired and validated by manufacturer with correspondent Lot number - Vero E6 (ATCC: CVCL_0574 - Lot 70034994), HEPG2 (ATCC: HB_8065 - Lot 70039681), Calu-3 (ATCC: HTB_55 Lot Lot 70035668) HeLa cells from VDG lab (HELA (ATCC: CVCL_0030)) were validated by profiling of human cell line using highly-polymorphic short tandem repeat loci (STRs) by Mycrosynth (date: 03.11.2016) https://www.microsynth.com/home-ch.html<br>HEK (ATCC: CRL_11268) and HEK293TphACE2-TMPRSS2 cells stable cell lines were verified in Fenwick, C. et al 2021 and 2022 by flow cytometry to ensure cell surface expression of human ACE2 and TMPRSS2 on 293T cells
Mycoplasma contamination	Cells were confirmed mycoplasma negative as tested on a trimestral basis using the MycoProbe Mycoplasma Detection Kit CUL001B
Commonly misidentified lines (See ICLAC register)	No commonly misidentified cell lines were used in this study

Animals and other research organisms

Policy information about studies involving animals; ARRIVE guidelines recommended for reporting animal research, and Sex and Gender in Research

Laboratory animals	All laboratory animals were housed at 22°C +/- 1°C, 55% (+/-10%) humidity; with 12 h light (from 7 to 19 h) and 12 h dark (from 19 h to 7 h)<br>Infection experiments - 20 female and 6 male eleven-week-old male and female K18-hAce2 C57BL/6j transgenic mice (strain: 2B6.Cg-Tg(K18-ACE2)2Prlmn/J) from The Jackson Laboratory were used. 4 female were used as uninfected controls whereas the remaining animals (16 females and 6 males) were used for infection.

	Control and DSS experiments - 6 Eight-weeks C57BL/6j male, rom The Jackson Laboratory, were used for Dextran Sulfate Sodium-induced colitis experiments (including controls).
Wild animals	No Wild animals where used in this study
Reporting on sex	Infection experiments - 20 female and 6 male eleven-week-old male and female K18-hAce2 C57BL/6j transgenic mice (strain: 2B6.Cg-Tg(K18-ACE2)2Prlmn/J) from The Jackson Laboratory were used. 4 female were used as uninfected controls whereas the remaining animals (16 females and 6 males) were used for infection.<br>Control and DSS experiments - 6 Eight-weeks C57BL/6j male, rom The Jackson Laboratory, were used for Dextran Sulfate Sodium-induced colitis experiments (including controls).
Field-collected samples	No field collected samples wher used in this study
Ethics oversight	Procedures were performed according to protocols approved by the Veterinary Authorities of the Canton Vaud and according to the Swiss Law (license VD3794A, EPFL).

Note that full information on the approval of the study protocol must also be provided in the manuscript.
